# The miR-561-5p/CX_3_CL1 Signaling Axis Regulates Pulmonary Metastasis in Hepatocellular Carcinoma Involving CX_3_CR1^+^ Natural Killer Cells Infiltration

**DOI:** 10.7150/thno.32543

**Published:** 2019-07-09

**Authors:** Er-Bao Chen, Zheng-Jun Zhou, Kun Xiao, Gui-Qi Zhu, Yi Yang, Biao Wang, Shao-Lai Zhou, Qing Chen, Dan Yin, Zheng Wang, Ying-Hong Shi, Dong-Mei Gao, Jie Chen, Yan Zhao, Wei-Zhong Wu, Jia Fan, Jian Zhou, Zhi Dai

**Affiliations:** 1 Liver Cancer Institute, Zhongshan Hospital, Fudan University, Shanghai 200032, China; State Key Laboratory of Genetic Engineering, Fudan University, Shanghai, 200032, China; 2 Key Laboratory of Carcinogenesis and Cancer Invasion, Fudan University, Ministry of Education, Shanghai, 200032, China; 3 Department of Medical Oncology, Zhongshan Hospital, Fudan University, Shanghai, 200032, China.; 4 Department of Liver Surgery and Transplantation, Zhongshan Hospital, Fudan University, Shanghai, 200032, China

**Keywords:** HCC, tumor microenvironment, NK cell, chemokine, CX_3_CL1

## Abstract

Natural killer (NK) cell can inhibit tumor initiation and regulates metastatic dissemination, acting as key mediators of the innate immune response. Intrinsic factors modulating NK cells infiltration and its anticancer activity remain poorly characterized. We investigated the roles of dysregulation of micro(mi)RNAs and NK cells in progression of hepatocellular carcinoma (HCC).

**Methods**: Small RNA sequencing were used to detect the miRNA profiles of tumor tissues from HCC patients with (n=14) or without (n=13) pulmonary metastasis and HCC cell lines with different pulmonary metastatic potentials. Chemokine expression profiling and bioinformatics were used to detect the downstream target of candidate target. In gain- and loss-of-function assays were used to investigate the role of miRNA in HCC progression. Different subsets of NK cells were isolated and used for chemotaxis and functional assays *in vivo* and *in vitro*. In situ hybridization and immunohistochemical analyses were performed to detect the expression of miRNA in tumor tissues from 242 HCC patients undergoing curative resection from 2010.

**Results**: Three miRNAs (miR-137, miR-149-5p, and miR-561-5p) were identified to be associated with pulmonary metastasis in patients with HCC. miR-561-5p was most highly overexpressed in metastatic HCC tissues and high-metastatic-potential HCC cell lines. In gain- and loss-of-function assays in a murine model, miR-561-5p promoted tumor growth and spread to the lungs. Yet, miR-561-5p did not appear to affect cellular proliferation and migration *in vitro*. Bioinformatics and chemokine expression profiling identified chemokine (C-X_3_-C motif) ligand 1 (CX_3_CL1) as a potential target of miR-561-5p. Furthermore, miR-561-5p promoted tumorigenesis and metastasis via CX_3_CL1-dependent regulation of CX_3_CR1^+^ NK cell infiltration and function. CX_3_CR1^+^ NK cells demonstrated stronger *in vivo* anti-metastatic activity relative to CX_3_CR1^-^ NK cells. CX_3_CL1 stimulated chemotactic migration and cytotoxicity in CX_3_CR1^+^ NK cells via STAT3 signaling. Blockade of CX_3_CL1, CX_3_CR1, or of pSTAT3 signaling pathways attenuated the antitumor responses. Clinical samples exhibited a negative correlation between miR-561-5p expression and levels of CX_3_CL1 and CX_3_CR1^+^ NK cells. High miR-561-5p abundance, low CX_3_CL1 levels, and low numbers of CX_3_CR1^+^ NK cells were associated with adverse prognosis.

**Conclusion**: We delineated a miR-561-5p/CX3CL1/NK cell axis that drives HCC metastasis and demonstrated that CX_3_CR1^+^ NK cells serve as potent antitumor therapeutic effectors.

## Introduction

Hepatocellular carcinoma (HCC), the most prevalent liver malignancy, is the third leading cause of cancer-related deaths. Most of the HCC cases and deaths, exceeding half a million annually, occur in developing countries, with China accounting for 50% of the cases 1. Various viral infections (including hepatitis B and C) and nonviral etiological factors, such as fatty liver disease, usually lead to diverse genetic change and eventually drive HCC development. Surgical resection and liver transplantation are common treatment options for HCC, the 5-year metastasis and recurrence rate after resection still exceeds 50% 2.

To metastasize, HCC cells must firstly fulfill certain tumorigenic functions and then move away from the primary site and enter the bloodstream through the vasculature. These malignant cells need to escape the killing of multiple immune cells, survive in the circulatory system and eventually form secondary cancer tissues in distant organs, such as lung, bone, and brain. Notably, lung is the most common metastatic organ. The tumor microenvironment contains a large number of cellular components and non-cellular components. Cellular components include tumor cells, immune cells, endothelial cells, fibroblasts and so on, while the non-cellular components include the extracellular matrix, cytokines, and chemokines 3. Lymphocytes within the tumor environment include T cells, B cells, and natural killer (NK) cells. Much research has focused on examining and attempting to reverse defects in CD8^+^T cells. However, NK cells, as key mediators of the innate immune response 4, represent one of the first blockers of tumor initiation and regulators of metastatic dissemination 5, 6. To date, NK cell-based antitumor immunotherapy approaches have not been tested in a clinical setting. Intrinsic factors modulating NK cells infiltration and anticancer activity remain poorly characterized. Understanding of these mechanisms can contribute to approaches that enhance NK cells-mediated tumor cell clearance.

Chemokines are associated with tumor-related inflammation. Chemokine signaling modulates various events associated with tumor progression, such as leukocyte recruitment, neo-angiogenesis, tumor cell proliferation, and metastasis. Fractalkine, also known as CX_3_C chemokine ligand 1(CX_3_CL1), is predominantly produced by the endothelium and binds only to the cognate chemokine (C-X_3_-C motif) receptor 1 (CX_3_CR1). CX_3_CR1 is a G-protein-coupled receptor expressed by CD8^+^ T lymphocytes 7, NK cells 8, and monocytes. The CX_3_CL1/CX_3_CR1 axis controls leukocyte migration 9, mediating chemotaxis and CX_3_CR1^+^ cell adherence. Because of its role in carcinogenesis and tumor progression, the CX_3_CL1/CX_3_CR1 axis has been explored as a candidate therapeutic target 10. However, CX_3_CL1 function in HCC and in NK cells-mediated eradication of cancer cells remains largely uncharacterized.

MicroRNAs (miRNAs) are small non-coding RNAs that participate in post-transcriptional regulation of gene expression 11. Expression of specific miRNAs, which can control a wide range of target genes, is often dysregulated in cancer cells 12, 13. Investigations of miRNAs in HCC metastasis can identify new prognostic biomarkers and therapeutic targets 14-16. Thus, we employed small RNA sequencing (smRNA-seq) to investigate the roles of miRNAs in HCC metastasis. We observed that elevated miR-561-5p levels are associated with pulmonary metastasis and a poor prognosis in HCC patients undergoing liver resection. Further studies revealed that miR-561-5p promoted HCC xenograft outgrowth and lung metastasis via the regulation of CX_3_CR1^+^NK cells infiltration. Mechanistically, miR-561-5p down-regulated CX_3_CL1 messenger RNA (mRNA) to reduce chemotaxis and function of CX_3_CR1^+^NK cells, but not of CX_3_CR1^-^NK cells. The CX_3_CR1^+^NK subset reduced the rate of pulmonary metastasis in a mouse model. Overall, our results demonstrated how the crosstalk between cancer cells and immune cells modulates HCC metastasis.

## Materials and Methods

### Human Subjects and Patient Evaluation

A total of 325 HCC patients were enrolled in this study. Samples collected between March and December of 2010 from 14 primary HCC patients with postoperative pulmonary metastasis (one sample excluded from analysis due to poor RNA quality) and 14 HCC patients without postoperative pulmonary metastasis were used for smRNA-seq. All samples were collected immediately after surgical resection, placed into liquid nitrogen and stored at -80°C. We also collected paired adjacent liver tissues of 13 primary specimens for smRNA-seq (three samples were not obtained, and one sample excluded from analysis due to poor RNA quality). Detailed clinicopathological features are listed in Supplementary Table S1. Samples from additional 55 HCC patients undergoing curative resection were collected for validation of associations between the candidate miRNA expression and prognosis.

Using SPSS software, we randomly selected 242 HCC patients undergoing curative resection in 2010 in the absence of pre-surgery anticancer therapy to construct tissue microarrays for expression analysis of miR-561-5p, CX_3_CL1, CX_3_CR1, and CD56. These patients were post-surgically monitored until July 7, 2015, with a median follow-up of 54 months (range: 1-66 months). The follow-up procedures were performed as described in our previous reports 17. The patients did not exhibit signs of distant metastasis.

Tumors were assessed using the World Health Organization histological classification, with differentiation graded using the Edmondson-Steiner system. Liver function was assessed using the Child-Pugh classification. The Barcelona-Clinic Liver Cancer and the tumor-node-metastasis classification systems were used to determine the disease stage. Overall survival (OS) was determined as the time that elapsed between surgery and death or the last observation point. Data of surviving patients were censored at the last follow-up. Time elapsing between the dates of the surgery and of any diagnosed intrahepatic or extrahepatic relapse was defined as time to recurrence (TTR). The protocols for the use of human subjects in this study were approved by the Research Ethics Committee of Zhongshan Hospital. Informed consent was obtained from all subjects.

### Cell Lines

The human hepatoma MHCC97L, MHCC97H, and HCCLM3 cell lines, demonstrating stepwise pulmonary metastatic potential, were previously developed at our institute. Low-metastatic-potential HepG2, PLC/PRF/5, SMCC-7721, and Huh7 cell lines were purchased from the Institute of Biochemistry and Cell Biology (Chinese Academy of Sciences, Shanghai, China). All cell lines were routinely maintained in Dulbecco's Modified Eagle Medium (GIBCO, NY, USA). Vectors used in this study, cell transfection procedures, NK cell isolation, chemotaxis assays, NK cytotoxicity assays, cell proliferation, and migration assays are described in the Supplementary Materials and Methods.

### Quantitative Polymerase Chain Reaction (qRT-PCR)

RNA isolation, reverse-transcription, and qRT- PCR are described in the Supplementary Materials and Methods.

### RNA and protein detection

Western blotting, enzyme-linked immunosorbent assays (ELISAs), immunohistochemistry procedures, and analysis, as well as in situ hybridization, are described in the Supplementary Materials and Methods.

### Animal Subjects

The study utilized 4- to 6-week-old male athymic BALB/c nu/nu mice (Shanghai Institute of Material Medicine, Chinese Academy of Sciences). The animals were maintained under specific-pathogen-free conditions. Humane animal care protocols were established according to the National Research Council Guide for the Care and Use of Laboratory Animals.

### smRNA-seq

Total RNA was isolated from approximately 10^6^ HepG2, PLC/PRF/5, MHCC97H, or HCCLM3 cells or from clinical samples using the TRIzol reagent (Invitrogen, Grand Island, NY). RNA quality and quantity were assessed using the 2100Bioanalyzer System (Agilent Technologies, Santa Clare, CA). The TruSeq Small RNA Library Prep Kit (Illumina) was used according to the manufacturer's protocol to prepare the smRNA-seq library using 1 μg of high- quality total RNA as the starting material. Sequencing was performed using the HiSeq2500 platform (Illumina) at GenergyBiotechnology (Shanghai) Co.Ltd. The FASTX Toolkit (Version 0.0.14) was used to filter out the adapter sequences. We quantified the reads mapping to known or novel miRNAs using miRBase v21 or miRDeep 2, respectively, and used the transcripts per kilobase million method to determine the normalized expression of each miRNA.

### Tumor Growth and Pulmonary Metastasis *In Vivo* Assays

Wild-type, knockdown, and overexpression (HepG2, HepG2-miR-561-5p, HepG2-shCX_3_CL1, HepG2-miR-561-5p-CX_3_CL1, HCCLM3, HCCLM3-anti-miR-561-5p, HCCLM3-CX_3_CL1, HCCLM3-anti-miR-561-5p-shCX_3_CL1) cells (5 × 10^6^) were suspended in 100 μL of a 1:1 mixture of serum-free Dulbecco's Modified Eagle Medium and Matrigel (BD Bioscience). The cell suspensions were injected subcutaneously into nude mice at the upper left flank region. After 4 weeks' injection, when subcutaneous tumors reached approximately 1cm in length, all tumors were peeled, sheared into 1mm^3^ volume and inserted into the livers of nude mice. Seven days following inoculation, animals in the NK depletion experimental group were intravenously injected with anti-Asialo- monosialotetrahexosylganglioside (GM1) antibody twice weekly. All animals were observed weekly and sacrificed 6 weeks post-inoculation. The IVIS Lumina K Series III system (PerkinElmer) was utilized to perform bioluminescence imaging, with radiance values normalized using the Living Image software. We observed the mice over 5 weeks for tumor formation. Tumor volume was calculated as follows: V=ab^2^/2, where V is the tumor volume in cm^3^, and a and b are the largest and smallest tumor diameters measured during necropsy, respectively. Following lung removal and embedding in paraffin, microscopy was used to determine the number of metastases per lung. For evaluating different function of CX_3_CR1^-^ and CX_3_CR1^+^NK cells *in vivo*, tail vein metastasis models of ten mice in each group were used. 1×10^6^ HCC cells were injected into mice, and then CX_3_CR1^-^ or CX_3_CR1^+^ NK cells were injected into mice. CX_3_CR1^-^ or CX_3_CR1^+^ NK cells were injected into the mice biweekly for 5 consecutive weeks after inoculation. The metastases were classified as grade I through grade IV based on the presence of ≤20, 20-50, 50-100, or >100 tumor cells at the maximal section of each metastatic lesion, respectively. CX_3_CR1^+^ or CX_3_CR1^-^ human allogenic NK cells (5 × 10^6^), purified from peripheral blood mononuclear cells from healthy donors using fluorescence-activated cell sorting, were injected intravenously.

## Results

### Overexpression of miR-561-5p is associated with HCC and HCC metastasis

**Figure 1:**
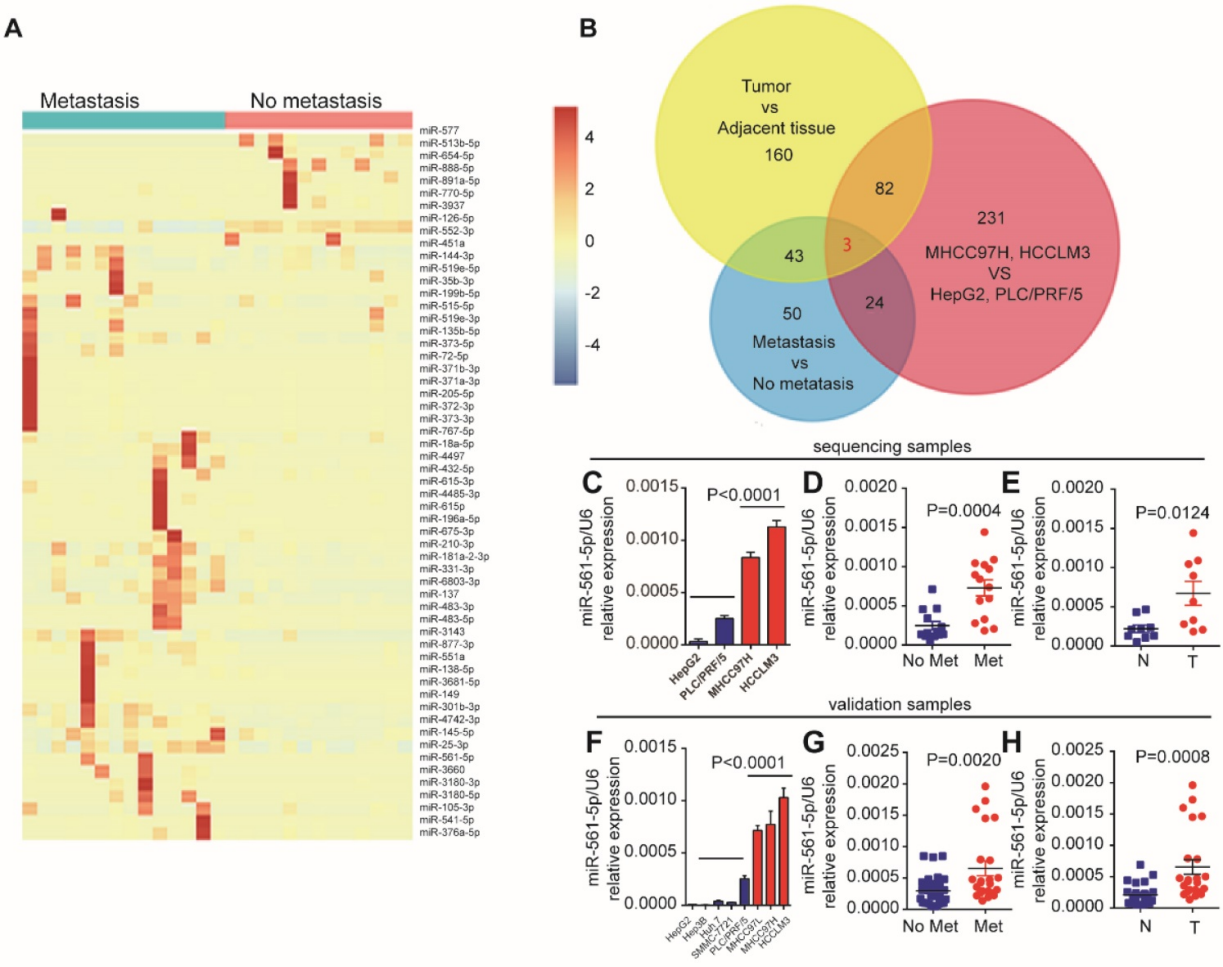
** MiR-561-5p expression was upregulated and positively correlated with pulmonary metastasis in HCCs.** (A) A heat map clustering of miRNAs with expression patterns that correlated with pulmonary metastasis in HCCs. (B) Venn diagrams showed that three miRNAs (miR-561-5p, miR-137, miR-149) were overlapped by the three groups. (C) Expression pattern of miR-561-5p in four HCC cell lines that correlated with metastatic potential in HepG2, PLC/PRF/5, MHCC97H, and HCCLM3. (D) Enhanced expression of miR-561-5p in HCCs with pulmonary metastasis (Met) versus HCCs without pulmonary metastasis (No Met) by qRT-PCR. (E) qRT-PCR revealed that miR-561-5p expression was significantly increased in the tumor (T), when compared to corresponding adjacent nontumor tissues (N). The data of (C, D, E) is generated from the sequencing samples by qRT-PCR. (F) Relative miR-561-5p levels in seven different HCC cell lines. (G) The patients suffering from metastasis (Met) exhibited higher miR-561-5p levels compared with patients without metastasis (No Met). (H) qRT-PCR revealed that the expression of miR-561-5p in HCCs was significantly increased in the tumor (T), when compared to corresponding adjacent nontumor tissues (N). The data of (F, G, H) was generated from eight HCC cell lines, and another cohort including 23 paired metastatic tumor and adjacent tissue sample (n=23), and cancer samples without metastasis (n=32)). Data shown are mean±SD from three independent experiments, each performed in triplicate. (*P < 0.05, **P<0.01, ***P < 0.001, ****P < 0.0001; Student's t-tests).

Lung metastases rarely occur in HCC patients that have undergone surgical resection. To identify miRNAs associated with HCC lung metastasis, we performed next-generation smRNA-seq to compare miRNA abundance patterns in primary human HCC with and without metastasis. We also performed smRNA-seq using high-metastatic-potential (MHCC97H and HCCLM3) and low-metastatic-potential (HepG2 and PLC/PRF/5) human HCC cell lines. Analysis of the clinical samples identified 57 differentially expressed (fold-change > 2, P < 0.01) miRNAs (Figure 1A). Moreover, 284 miRNAs demonstrated metastatic potential-dependent abundance levels in the HCC cell lines. Further analysis of tumors and adjacent non-tumor tissues identified 321 differentially expressed miRNAs (Supplementary Figure S1A, Supplementary Excel 1). Combination of these bioinformatics analyses suggested that expression of miR-137, miR-149-5p, and miR-561-5p, which was confirmed by qRT-PCR, correlates with HCC progression and lung metastasis (Figure 1B). As miR-561-5p demonstrated the most substantial changes in abundance (Figure 1C-E, and Supplementary Figure S1B-C), we subsequently sought to explore the role of miR-561-5p in HCC lung metastasis.

First, we evaluated the relationship between endogenous miR-561-5p levels and the metastatic potential of HCC lines. Cell lines with high- metastatic-potential exhibited elevated expression of miR-561-5p relative to non-metastatic cell lines, indicating the miR-561-5p were positively correlated with increasing metastatic potential (Figure 1F). Next, using qRT-PCR, we measured the expression of miR-561-5p in paired tumor and adjacent tissue samples from HCC cohorts with (n=23) and without metastasis (n=32). Highest miR-561-5p levels were observed in tumors with metastasis, whereas lowest levels were detected in the adjacent non-tumor tissues (Figure 1G-H). Taken together, these findings prompted us to investigate the mechanisms by which miR-561-5p contributes to HCC metastasis.

### miR-561-5p promotes tumor progression and lung metastasis *in vivo*, without affecting HCC cell proliferation and invasion *in vitro*

To further determine the effects of miR-561-5p in HCC, miR-561-5p levels in HCC cell lines were modulated using miR-561-5p or anti-miR-561-5p lentiviral vectors. Altered miR-561-5p levels were confirmed via qRT-PCR (Supplementary Figure S2A). Down-regulation of miR-561-5p in HCCLM3 and MHCC97H cells did not affect proliferation rates. Proliferation rates of miR-561-5p-overexpressing HepG2 and PLC/PRF/5 cells were similar to those of control cells (Supplementary Figure S2B). Modulation of miR-561-5p levels also had no effect on migration and invasion of HCC cells (Supplementary Figure S2C- D).

**Figure 2:**
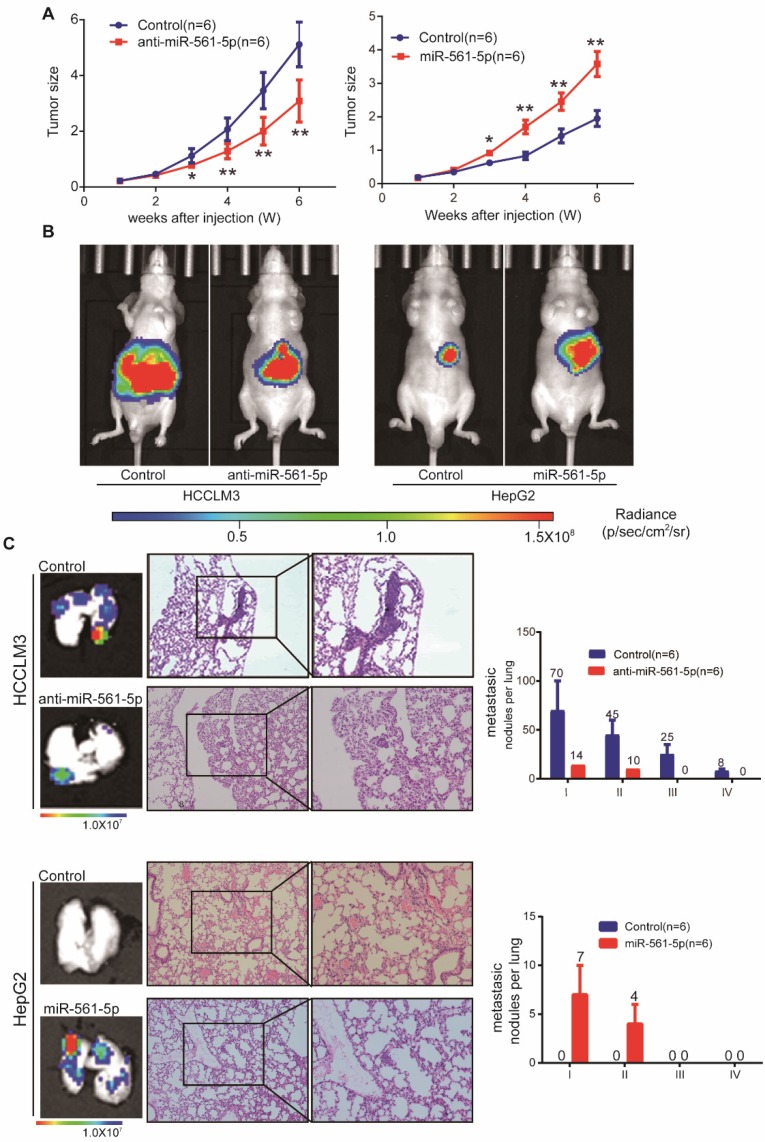
** MiR-561-5p enhances tumor outgrowth and pulmonary metastasis of xenograft tumors from two human HCC cell lines.** (A) Growth curves of tumors in xenograft nude mouse models are shown compared with empty vector. (B) Representative bioluminescence images of mice show subcutaneous tumors at day 42 after inoculation of HCC cells. The color scale bar depicts the photon flux emitted from these mice. (C) Representative images of H&E staining of metastatic nodules in lungs from different animal groups (left panel). The grades of metastases in each group are indicated (right panel). Data shown are mean±SD (n=6). (*P<0.05; **P<0.01, Student's t-tests).

To assess whether miR-561-5p affects tumor growth and metastasis *in vivo*, HCC cells harboring different miR-561-5p levels were injected into nude mice. The tumor size of the HCCLM3-derived xenografts (5.12 ± 0.73 cm^3^) was significantly larger than that of xenografts derived from HCCLM3-anti- miR-561-5p cells (3.09 ± 0.69 cm^3^). Similarly, the average tumor size in the HepG2-miR-561-5p group was significantly greater than that of the HepG2-derived tumors (3.58 ± 0.85 cm^3^ vs 1.95 ± 0.54 cm^3^; Figure 2A). These results were confirmed by bioluminescence imaging (Figure 2B). Whereas pulmonary metastases were observed in all animals in the HCCLM3 group, <20% of the mice (1/6, P=0.015) exhibited metastases in the HCCLM3-anti-miR-561-5p group. Animals in the HCCLM3 group also harbored more metastatic nodules of each grade (Figure 2C). Likewise, pulmonary metastasis was observed in 83.3% (5/6) of the animals in the HepG2-miR-561-5p group, but was not detected in the HepG2 group (Figure 2C, P=0.015). These finding demonstrated that miR-561-5p promotes HCC growth and pulmonary metastasis *in vivo*.

### CX_3_CL1 is a direct downstream target of miR-561-5p

**Figure 3:**
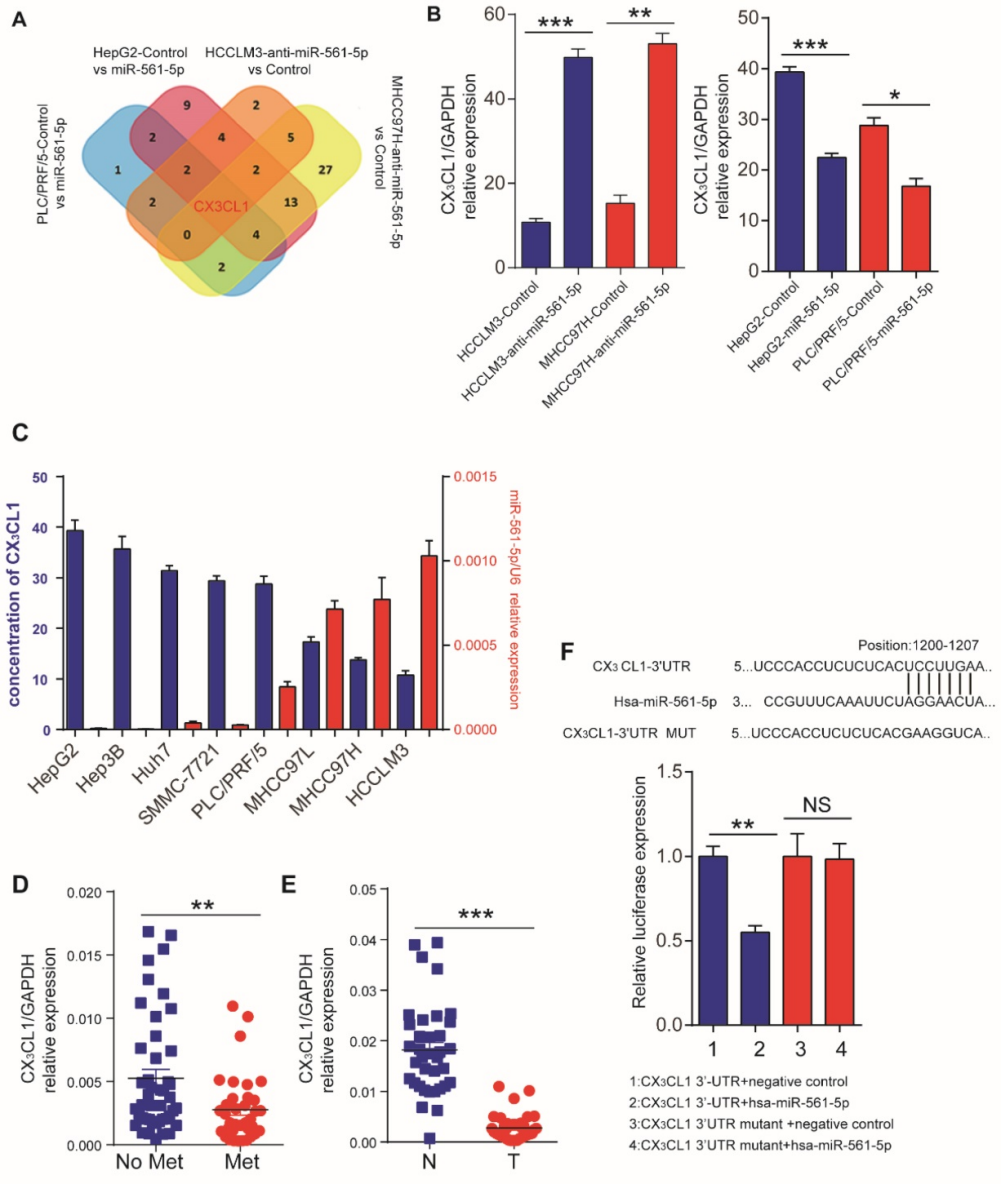
** Identification of CX_3_CL1 as a direct downstream target of miR-561-5p.** (A) Venn diagrams showing the number of genes identified as potential targets of miR-561-5p according to four groupings: (1) upregulated cytokines in HCCLM3/MHCC97H cells after transfection with anti-miR-561-5p; (2) downregulated cytokines in HepG2/PLC/PRF/5 cells after transfection with miR-561-5p. (B) qRT-PCR validated that knockdown of miR-561-5p in HCCLM3/MHCC97H cells, while miR-561-5p was forced expression in HepG2/PLC/PFR/5 cells. (C) Elisa showed that the expression of CX3CL1 was negatively correlated with the expression of miR-561-5p. (D) Expression of miR-561-5p in HCC tissues with (Met) or without pulmonary metastasis (No Met) was determined by qRT-PCR. (E) Expression of CX_3_CL1 in tumor tissues (T) was significantly decreased when compared to corresponding adjacent nontumor tissues (N). (F) Sequences of hsa-miR-561-5p and its potential binding site at the 3'UTRs of CX_3_CL1 are shown and the nucleotides mutated in CX_3_CL1 3'UTR mutant (upper panel). miR-561-5p significantly suppressed the luciferase activity of CX_3_CL1 containing a wild-type 3'-UTR, but showed no effect on the activity of CX_3_CL1 with a mutant 3'-UTR. (lower panel). Luciferase activity was normalized to the activity of β-galactosidase. Data shown are mean±SD from three independent experiments, each performed in triplicate. (*P<0.05; **P<0.01, Student's t-tests).

The discrepancy between the *in vitro* and *in vivo* results indicated that a cell nonautonomous mechanism may be responsible for miR-561-5p effects. One such mechanism involves the regulation of cytokines to modulate the tumor microenvironment 18. Thus, we assessed the effect of miR-561-5p on cytokine expression by testing HCCLM3 and MHCC97H cells transfected with anti-miR-561-5p and HepG2 and PLC/PRF/5 cells transfected with miR-561-5p. CX_3_CL1 was the only chemokine that demonstrated consistent inverse correlation with miR-561-5p in all experimental groups (Figure 3A, Supplementary Table S2,). ELISA confirmed elevated CX_3_CL1 levels following miR-561-5p knockdown in HCCLM3 and MHCC97H cells. In contrast, overexpression of miR-561-5p markedly reduced CX_3_CL1 expression in HepG2 and PLC/PRF/5 cells (Figure 3B).We also observed that basal levels of CX_3_CL1 negatively correlated with miR-561-5p levels in eight HCC cell lines (Figure 3C).Moreover, the lowest levels of CX_3_CL1 expression were observed in HCC with metastasis, whereas the highest levels were detected in the paired non-tumor tissues (Figure 3D-E).

TargetScan (http://www.targetscan.org) and miRDB (http://mirdb.org) bioinformatics analyses predicted CX_3_CL1 as a potential target of miR-561-5p. The putative binding sequence of miR-561-5p was predicted to be located within the 3' untranslated region (UTR) of the CX_3_CL1 mRNA (Figure 3F). Expression of miR-561-5p effectively reduced luciferase activity of the CX_3_CL1 reporter construct. This effect was abrogated via seed sequence mutations in predicted miR-561-5p binding sites. Taken together, these experiments confirmed a direct interaction between miR-561-5p and the 3'-UTR of CX_3_CXL1 mRNA *in vitro* and *in vivo*.

### CX_3_CL1 triggers chemotactic migration and cytotoxicity of NK cells via signal transducer and activator of transcription 3 (STAT3) signaling

**Figure 4:**
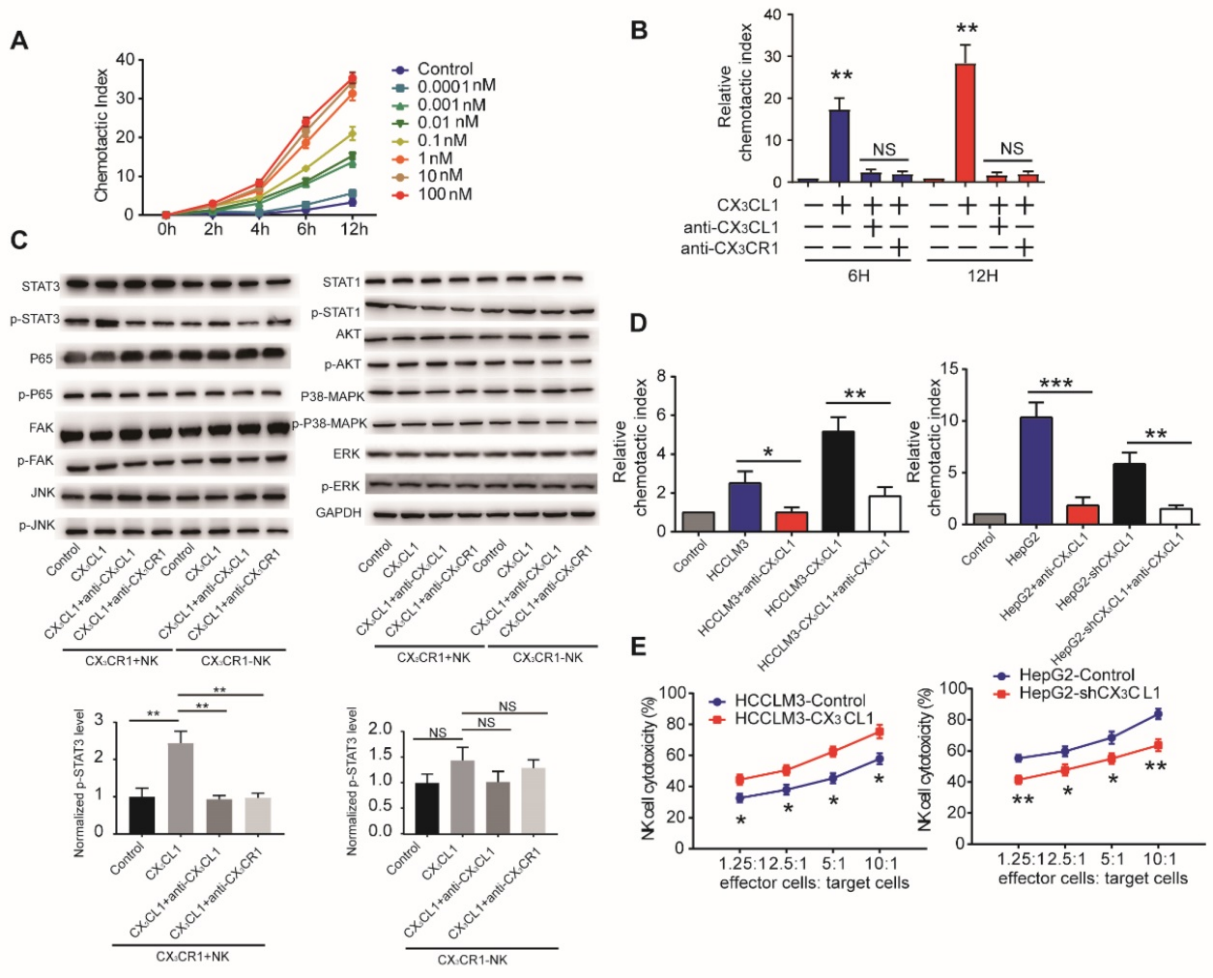
** CX_3_CL1 selectively activates STAT3 signaling in CX_3_CR1+NK cells instead of CX_3_CR1^-^NK cells.** (A)CX_3_CL1 induced concentration-dependent chemotactic migration of NK cells *in vitro* within the range of 0.0001-100 nM, *P<0.05; **P<0.01 compared with control. (B) Anti-CX_3_CL1 or anti-CX_3_CR1 abolished the chemotactic migration of NK cells by CX_3_CL1. (C) Western blotting showed that CX_3_CL1 treatment caused s significant increase in the phosphorylation level of STAT3. (D) Chemotactic index of NK cells was measured following stimulation with conditioned medium (CM) derived from different HCC cells compared with other groups. (E) NK cells cytotoxicity showed that CX_3_CL1 stimulates the NK cytotoxicity by LDH assays. The chemotactic index represents the number of cells that migrated toward the chemoattractant in the lower chamber compared with the negative control. Data shown are mean±SD from three independent experiments, each performed in triplicate. (*P<0.05; **P<0.01, Student's t-tests).

CX_3_CL1 can recruit CX_3_CR1^+^ inflammatory cells 19. Therefore, we tested the effect of recombinant CX_3_CL1 on the migration of monocytes, T cells, dendritic cells, and NK cells isolated from HCC patients. Only NK cells exhibited CX_3_CL1 concentration- dependent chemotactic migration (Figure 4A, Supplementary Figure S3A). This response was reversed by an anti-CX_3_CL1 antibody and attenuated using an anti-CX_3_CR1 antibody (Figure 4B).

Till now, it is still not clarified which pathways were activated in CX_3_CR1^+^NK cells. In our study, we investigated some critical classical pathways for NK cells.

We first performed western blotting analysis of NK cells in the presence or absence of 100 nM CX3CL1 to assess potential pathways associated with the CX_3_CL1-mediated effects on NK cells. We observed that CX_3_CL1 led to the increased phosphorylation of STAT3, but did not affect p65, FAK, JNK, STAT1, AKT, p38-MAPK, or ERK phosphorylation in CX_3_CR1^+^NK cells. Furthermore, blocking CX_3_CL1 or CX_3_CR1 by anti-CX_3_CL1 or anti-CX_3_CR1 antibody in CX_3_CR1^+^NK cells caused a significant decrease in the phosphorylation levels of STAT3 compared with CX_3_CL1 group. These results revealed that the effect of CX_3_CL1 on STAT3 phosphorylation in CX_3_CR1^+^NK cells was dependent on the CX_3_CL1 receptor, CX_3_CR1 (Figure 4C, Supplementary Figure S3B).

Next, we assessed whether HCC cells use CX_3_CL1 to recruit NK cells *in vitro*. Conditioned medium from high CX_3_CL1 expressers (HCCLM3- CX_3_CL1 and HepG2-Control) significantly increased the migration of NK cells relative to that from low CX_3_CL1 expressers (HCCLM3-Control and HepG2- shCX_3_CL1; Figure 4D). Anti-CX_3_CL1 antibodies eliminated the chemotactic migratory effect of the conditioned medium. Then we assessed the effect of CX_3_CL1 levels on cytolytic activity of NK cells co-cultured with different HCC cell lines. Co-culture with HCCLM3-CX_3_CL1 cells enhanced the cytolytic activity of NK cells, whereas the presence of HepG2- shCX_3_CL1 cells reduced the cytolytic activities of NK cells (Figure 4E). These data indicated that CX_3_CL1 can directly chemoattract NK cells and enhance their cytotoxicity via STAT3 phosphorylation.

### Overexpression of miR-561-5p down-regulates CX_3_CL1 and promotes tumor growth and metastasis via suppression of NK cells infiltration

**Figure 5:**
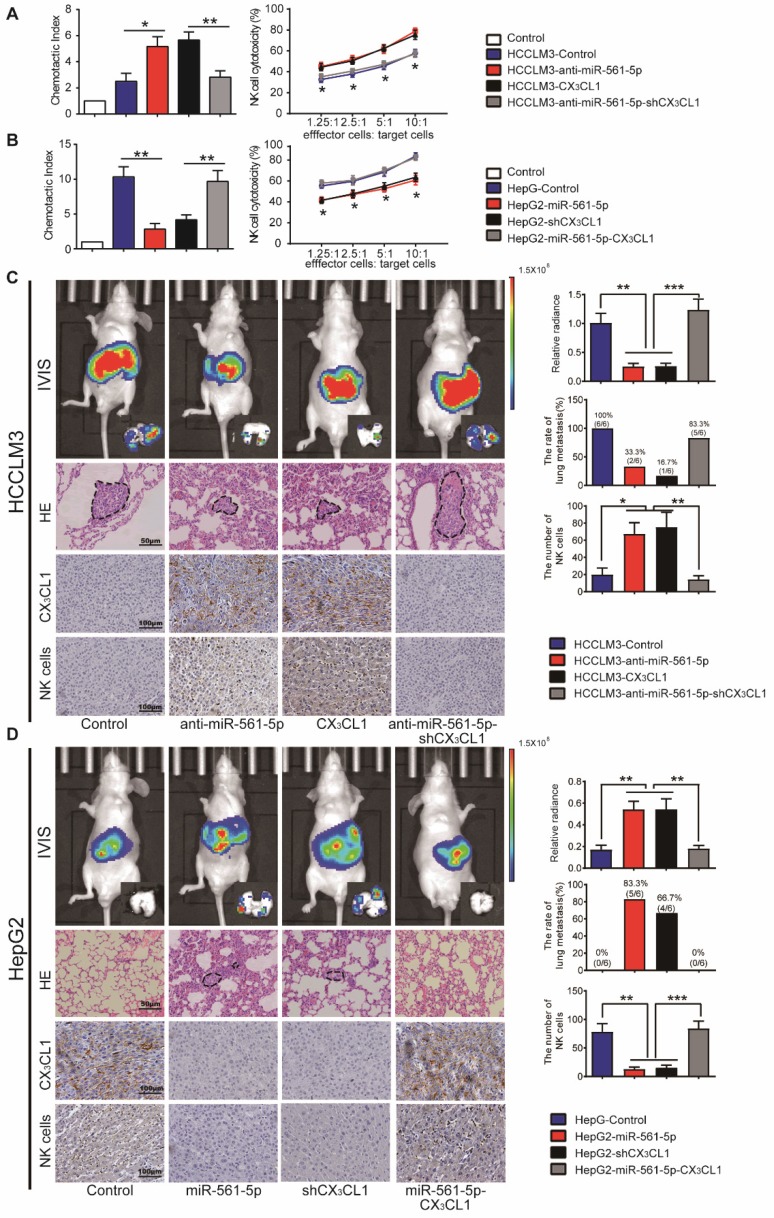
** MiR-561-5p-CX_3_CL1 signaling mediates NK infiltration and modulates HCC progression and metastasis.** (A and B) miR-561-5p inhibits NK migration by targeting CX_3_CL1 in HCCLM3 cells (A) and HepG2 cells (B). (C and D) Representative bioluminescence images of animals, H&E staining of metastatic nodules in lung, and immunostaining of CX_3_CL1 and NK cells in tumor sample serial sections from xenograft nude mouse models derived from HCCLM3 (C) and HepG2 (D) cells are shown. Data shown are mean±SD from three independent experiments, each performed in triplicate. (*P<0.05, **P<0.01, ***P<0.001, Student's t-tests).

We next assessed whether the effect of miR- 561-5p on HCC progression is negatively correlated with CX_3_CL1-induced NK cells infiltration. Knockdown of miR-561-5p or overexpression of CX_3_CL1 enhanced the migratory effect of the HCCLM3 cell conditioned medium on NK cells. Secondary knockdown of CX_3_CL1 eliminated the anti-miR-561- 5p effect. Overexpression of miR-561-5p or CX3CL1 knockdown reduced chemotaxis of NK cells in HepG2 cell conditioned medium. However, CX_3_CL1 overexpression reversed the miR-561-5p effects (Figure 5A-B). These findings indicated that HCC cells use miR-561-5p to inhibit NK cell migration via down-regulation of CX_3_CL1.

To further assess the inhibitory function of CX_3_CL1 in HCC development, we modulated CX_3_CL1 levels in HCC cells. In a manner similar to miR-561-5p modulation, manipulation of CX_3_CL1 levels did not affect the phenotype of HCC cells *in vitro* (data not shown). However, *in vivo*, CX_3_CL1 reduced tumorigenesis and pulmonary metastasis, with CX_3_CL1 suppression promoting tumor development (Figure 5C-D). NK cells infiltrated the HCCLM3-derived xenografts at a rate lower than the HCCLM3-CX_3_CL1 xenografts. Likewise, HepG2-derived xenografts contained higher numbers of infiltrating NK cells relative to the HepG2-shCX_3_CL1 group (Figure 5C-D). On the other hand, there was no difference in the numbers of monocyte and myeloid-derived suppressor cells (Supplementary Figure S4A-B).

We subsequently assessed how the miR-561-5p/ CX_3_CL1/NK cells axis regulates HCC progression and pulmonary metastasis. CD56 is a specific surface marker of NK cells. We counted the number of positive staining cell with CD56 in each group. CX_3_CL1 overexpression or miR-561-5p knockdown in HCCLM3 cells significantly suppressed tumor volume, reduced the rate of lung metastasis, and enhanced NK cells infiltration. Knockdown of CX_3_CL1 countered the effects of anti-miR-561-5p. Furthermore, overexpression of CX_3_CL1 rescued the HepG2-miR-561-5p phenotype (Figure 5D), indicating that miR-561-5p drives HCC development by suppressing CX_3_CL1-induced NK cells infiltration.

### CX_3_CR1^+^ NK cells suppress HCC progression in a murine model

**Figure 6:**
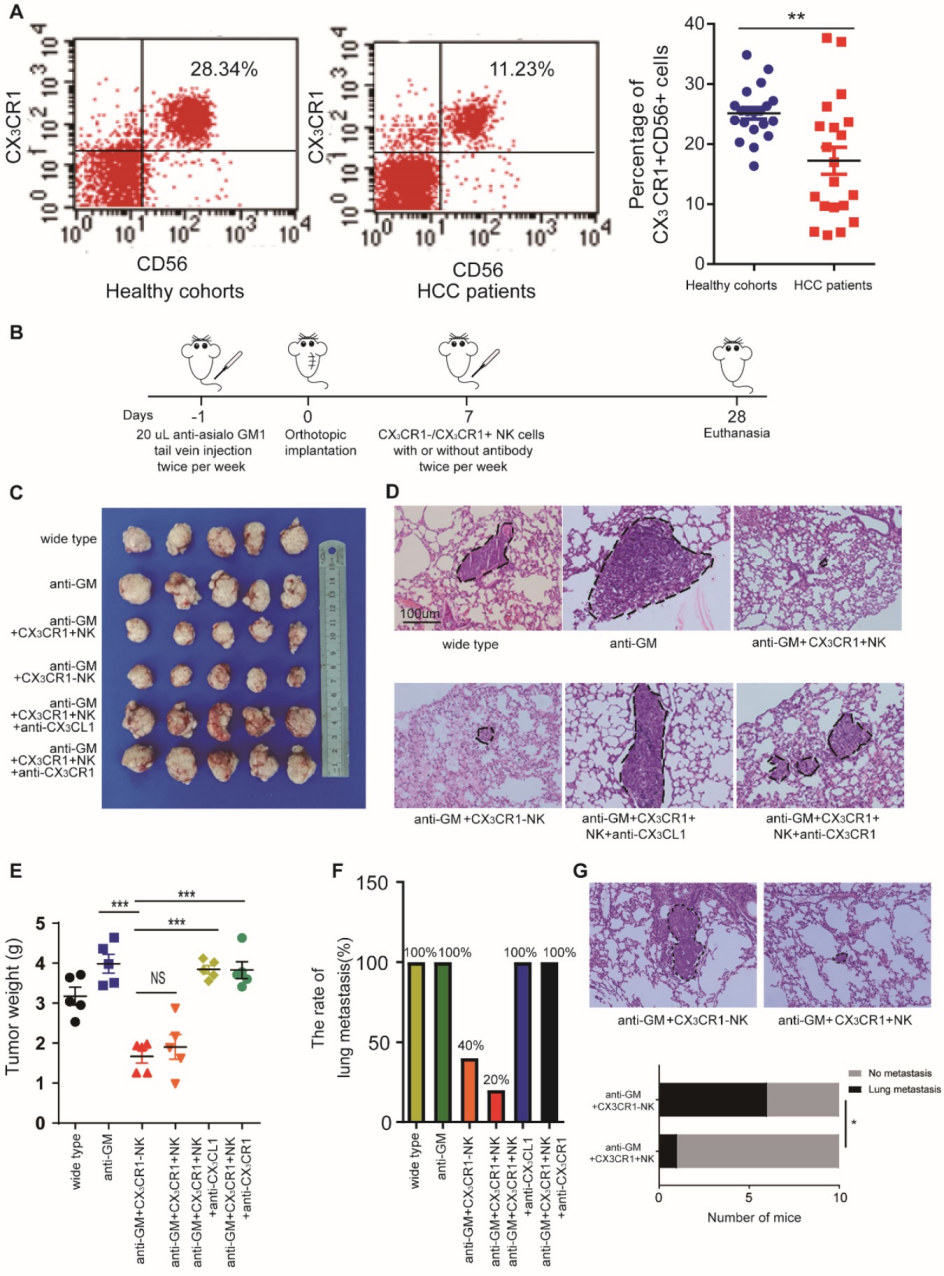
** CX_3_CR1^+^NK cells activated by CX_3_CL1 control HCC pulmonary metastasis in mice models.** (A) Flow cytometry plots showed CX_3_CR1^+^NK cells are abundant in healthy donors' peripheral blood compared with HCC patients. (B) The detailed model diagram was shown. In this study, we pretreated nude mice with 20 uL anti-asialo GM antibodies injected into the tail vein per mouse the day before xenotransplantation, and the antibodies were injected twice a week. We began to inject immune cells and/or antibodies into the tail vein to treat nude mice one week after xenotransplantation, and all nude mice were euthanized after 4 weeks for analysis. Injection of CX_3_CR1^+^NK cells or CX_3_CR1^-^NK cells resulted in decreased tumor growth (C, E) and pulmonary metastasis (n=5) (D, F), and significantly impaired by co-injection with anti-CX_3_CL1 or anti-CX_3_CR1 antibodies. Circles indicate cancer metastasis (magnification, 200X). Scale bar, 100 μm. (G) The pulmonary metastasis of mice with CX_3_CR1^+^NK cells showed a smaller rate that that of mice with CX3CR1^-^NK cells (1/10 vs 6/10). (**P<0.01, ***P<0.001. Student's t-tests, Fisher exact tests).

Based on the above results, we hypothesized that miR-561-5p suppresses CX3CL1 levels, thus reducing the infiltration of NK cells. CX_3_CR1^+^NK cells, harboring a CX_3_C chemokine receptor, play a critical role in CX_3_CL1-induced NK cell migration. Using flow cytometry, we assessed the prevalence of CX_3_CR1^+^NK cells in 20 fresh HCC patients' peripheral blood and 20 fresh healthy control samples (Figure 6A). The results showed CX_3_CR1^+^NK cells are abundant in healthy donors compared with HCC patients.

To investigate the effect of CX_3_CR1^+^ NK cells on HCC growth and metastasis *in vivo*, we administered the NK cell-depleting anti-Asialo-GM1 antibody to nude xenograft mice and determined their response to CX_3_CR1^+^NK cells. The mice were treated with either CX_3_CR1^+^ or CX_3_CR1^-^NK cells twice a week for three weeks (Figure 6B). The average tumor size and pulmonary metastasis rate were decreased in mice treated with CX_3_CR1^+^ NK cells alone relative to those treated with CX_3_CR1^+^NK cells combined with an anti-CX_3_CL1 antibody or an anti-CX_3_CR1 antibody (Figure 6C-E). Likewise, the pulmonary metastasis rate in mice treated with CX_3_CR1^+^ NK cells was markedly decreased relative to that in mice treated with CX_3_CR1^-^NK cells (Figure 6F). To compare the functional difference on circulating HCC cells metastasis between CX_3_CR1^+^NK cells group and CX_3_CR1^-^NK cells group, we designed new experiments. We injected HCC cells and then we injected CX_3_CR1^+^ or CX_3_CR1^-^ NK cells in ten mice each group by tail vein injection lung metastasis model. *In vivo* study showed that injection of CX_3_CL1^+^NK following HCC cells resulted in significantly decreased pulmonary metastasis compared HCC cells with injection of CX_3_CR1^-^NK cells (1/10 vs 6/10) (P<0.05, Figure 6G). The results suggested that the CX_3_CL1/CX_3_CR1 axis might be responsible for the negligible therapeutic effect against HCC in nude mice, perhaps due to enhanced cytotoxicity of CX_3_CR1^+^NK cells.

### miR-561-5p, CX_3_CL1, and CX_3_CR1^+^NK cells predict HCC patient prognosis

**Figure 7:**
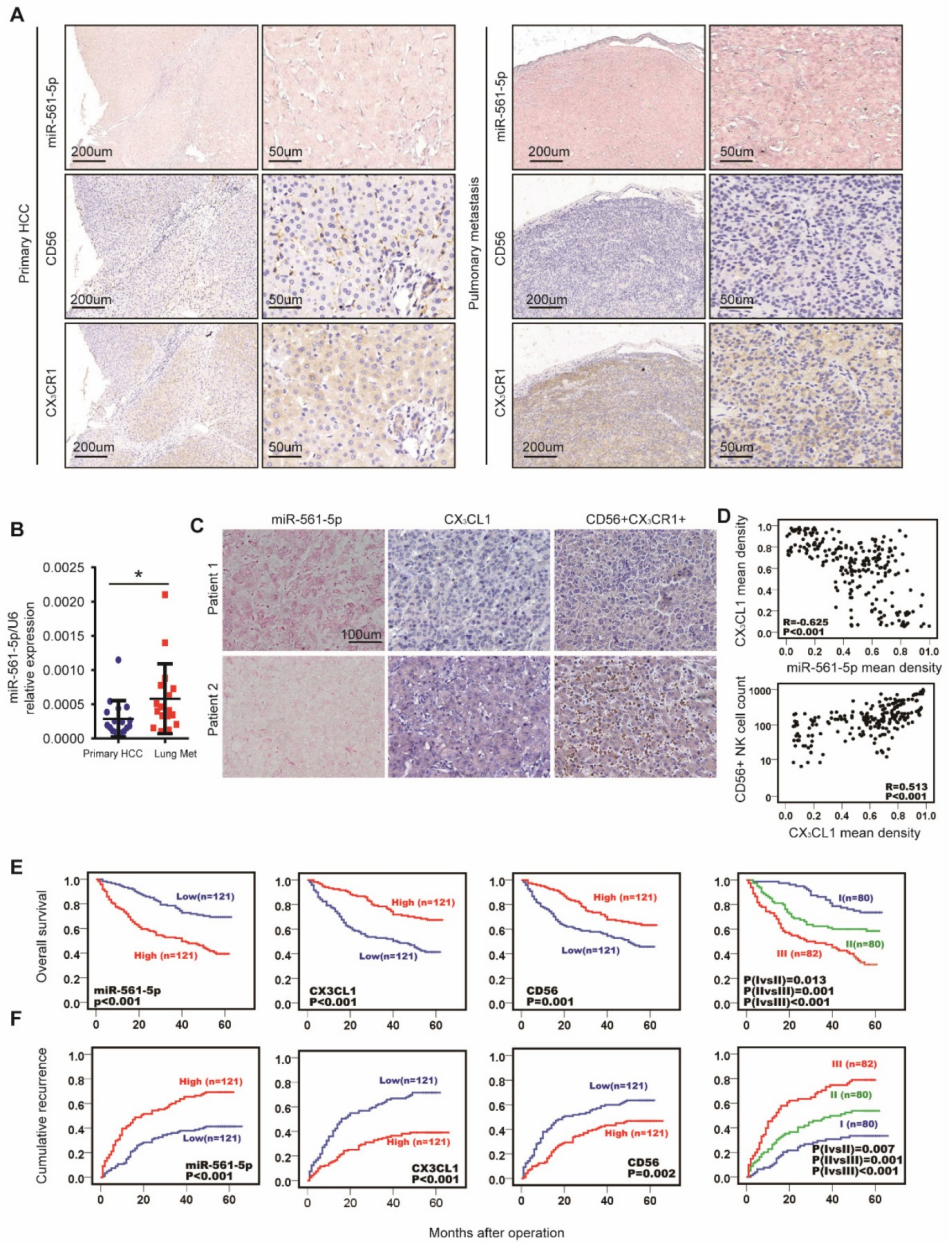
** MiR-561-5p-CX_3_CL1-CX_3_CR1+NK axis displayed the strong prognostic value in HCC patients**. (A) Levels of miR-561-5p, CX_3_CL1 and CX_3_CR1^+^ in representative HCCs primary tumors (left panel) and corresponding pulmonary tumors (right panel). Scale bars, 50x, 200 μm, 200x, 50 μm. (B) qRT-PCR revealed that miR-561-5p expression was significantly increased in pulmonary metastasis (Lung Met), when compared to corresponding primary tumors (Primary HCC). (C) Levels of miR-561-5p, CX3CL1 and CX_3_CR1^+^NK in representative HCC patients in TMA are shown. Patient 1 had high-level expression of miR-561-5p and low-level expression of CX_3_CL1 and CX_3_CR1^+^NK, while patient 2 had low-level expression of miR-561-5p and high expression of both CX_3_CL1 and CX_3_CR1^+^NK. (scale bar, 100 μm) (D) Scatterplot depicts a significant inverse correlation between miR-561-5p and CX_3_CL1 and a significant positive correlation between CX_3_CL1 and CX_3_CR1^+^NKs in cancerous tissues. (E-F) Prognostic values of miR-561-5p, CX_3_CL1, and CX_3_CR1^+^NK by Kaplan-Meier analysis. I, miR-561-5p^high^/CX_3_CL1^low^/CX_3_CR1^+^ NK^low^; III, miR-561-5p^low^/CX_3_CL1^high^/CX_3_CR1^+^ NK^high^; II, others. Data are representative of three independent experiments. Scale bar, 100 μm. (*P<0.05, **P<0.01, ***P<0.001. Student's t-tests; log-rank test; Cox proportional hazard regression models).

Colonization of the lung is a crucial step in HCC pulmonary metastasis. The above results indicated that the miR-561-5p/CX_3_CL1/NK cell axis promotes HCC growth and lung metastasis. To further explore the clinical relevance of this axis, we performed in situ hybridization to determine miR-561-5p localization, in combination with immuno-staining of CX_3_CR1^+^NK cells, on serial sections of clinical samples from primary cancers and paired lung metastases. Tumor cells and surrounding fibroblasts exhibited miR-561- 5p expression (Figure 7A). We observed miR-561-5p expression was higher in the metastatic lung niche than that in primary cancers, with fewer CX_3_CR1^+^NK cells detected in the metastatic lung niche (Figure 7A). To confirm the expression of miR-561-5p in HCC tissue and lung metastatic tissue sample, RT-PCR revealed that the expression of miR-561-5p in significantly decreased in primary tumors compared to the corresponding lung metastatic tissues (Figure 7B). These data underscored the hypothesis that high levels of miR-561-5p in primary HCC and metastases suppress NK cells-mediated eradication of cancer cells and promote tumor dissemination and colonization of the lung.

To evaluate the clinical prognostic significance of miR-561-5p, CX_3_CL1, and CX3CR1^+^NK cells, we assessed the expression of miR-561-5p, CX_3_CL1, CX_3_CR1, and CD56 in primary tumors from a distinct 242-patient HCC cohort (Figure 7C-D). Levels of miR-561-5p inversely correlated with survival rates, as determined using the Kaplan-Meier analysis. The high-level miR-561-5p HCC patients demonstrated the poorest prognosis at 1, 3, and 5 years and exhibited higher cumulative recurrence rates than low-level miR-561-5p patients (Figure 7E-F). In contrast, CX_3_CL1 levels positively correlated with survival rates and negatively correlated with recurrence rates (Figure 7E-F). Detection of CX_3_CR1^+^NK cells exhibited a positive correlation with OS (P<0.029) and TTR (P<0.001). Moreover, low-level miR-561-5p expression correlated with higher CX_3_CL1 abundance and more NK cells infiltration in HCC patients (Figure 7C-D). To assess the combined prognostic value of these factors, we assigned patients to the following three groups: group I comprised patients with low miR-561-5p, high CX_3_CL1, and high CX_3_CR1^+^NK cells levels; group III comprised patients with high miR-561-5p, low CX_3_CL1, and low CX_3_CR1^+^NK cells levels; group II comprised the remaining patients. Group I OS values were higher than those of group II, with the lowest values observed for group III (Figure 7E-F). Group I cumulative recurrence rates were substantially lower than those observed for individuals in groups II and III (Figure 7F). Overall, miR-561-5p levels, CX_3_CL1 abundance, CX_3_CR1^+^NK cells infiltration, and the co-index were found to be independent prognostic factors of HCC OS and TTR (Table 1).

## Discussion

**Figure 8:**
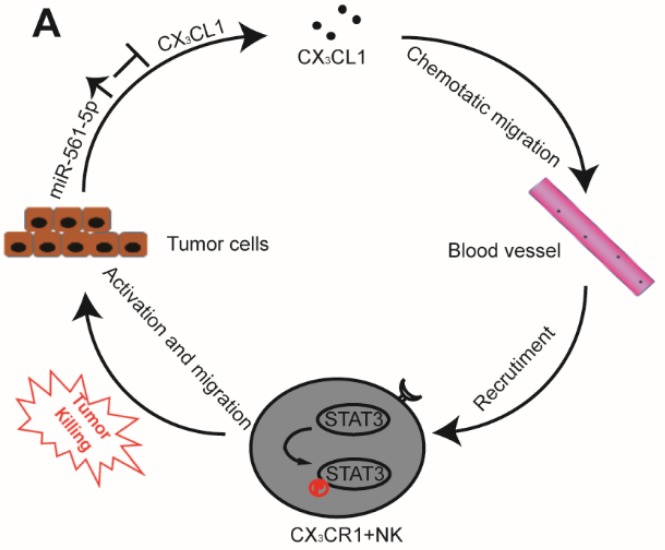
A Proposed model illustrates the role of the miR-561-5p-CX_3_CL1- CX_3_CR1^+^NK loop in the regulation of HCC metastasis.

The interplay between tumor cells and their microenvironment influences a variety of processes, including tumor invasion, metastasis, and response to therapy. NK cells, a major component of the tumor microenvironment, constitute 30-40% of all intrahepatic lymphocytes and exhibit antitumor functions 20. We identified a novel signaling pathway in which miR-561-5p alters the tumor microenvironment by suppressing its target CX3CL1 and consequently blocking the recruitment and infiltration of NK cells, thereby allowing HCC cells to evade NK cell cytotoxicity, proliferate, and metastasize to the lungs (Figure 8).

To our knowledge, this is the first work to report a positive correlation between miR-561-5p levels and pulmonary metastasis, establishing miR-561-5p as both a risk factor and a prognostic marker in HCC metastasis. Overexpression of miR-561-5p in HCC patients corresponded to lung metastasis and poor prognosis, underscoring its cancer-promoting role in HCC progression. In contrast to other miRNAs implicated in HCC progression, miR-561-5p promoted tumor progression by influencing tumor microenvironment rather than tumor cell intrinsic activity, and stimulated tumor growth and pulmonary metastasis in animal models. A study of 242 patients revealed that the level of miR-561-5p represents a new and meaningful molecular marker to predict HCC patient prognosis. This pro-metastatic role of miR-561-5p was found to be based on its regulation of CX_3_CR1^+^NK cell function via its target CX_3_CL1.

Identification of miR-561-5p was somewhat surprising, as very little is known about its role in cancer 21.To determine the role of miR-561-5p, we speculated that it can modulate cytokine or chemokine levels to reshape the tumor microenvironment to favor metastasis 22. CX_3_CL1 mRNA was identified as the direct downstream target of miR-561-5p, with miR-561-5p overexpression in HCC patients reducing CX_3_CL1 levels, indicating that the crosstalk between cancer cells and their associated stroma cells is potentially regulated by CX_3_CL1. Furthermore, our results confirmed that the main resource of CX_3_CL1 is tumor cells, not endothelial cells or other stromal cells (Supplementary Figure S5A-B). A dual luciferase reporter assay and rescue experiments confirmed CX_3_CL1-dependent regulation of HCC progression by miR-561-5p.

CX_3_CL1, which signals through CX_3_CR1, can recruit and activate NK cells via upregulation of several key proteins. However, to date, the function of this chemokine in tumorigenesis has remained poorly characterized. CX_3_CL1 has been shown to robustly affect the chemotactic movement of NK cells 23, 24. Presently, we demonstrated that CX_3_CL1 stimulates CX_3_CR1^+^NK cell chemotactic migration and cytotoxicity, a response abolished by anti-CX_3_CL1 or anti-CX_3_CR1 antibodies. The antitumor and anti-metastatic effects of CX_3_CL1 were driven by NK cell recruitment and infiltration, as revealed by *in vivo* studies. Considering that CX_3_CR1 is expressed in T cells as well as NK cells in intact humans and mice, T cells may be responding to the chemokine CX_3_CL1. Our *in vitro* experiments only observed the low chemotactic effects of CX3CL1 on T cells, compared with those on NK cells. In addition, we immune-stained CD8+T cells in the TMA and found that the level of miR-561-5p was not associated with the number of CD8+T cells (P=0.621, Supplementary Figure S6A-B). Aoyama et al 25 described CX_3_CL1- CX_3_CR1 interaction could prevent liver inflammation and fibrosis. This observation, which contradicts our observation, may stem from the difference between the specimens and the isolated cells. In his study, NK cells were not isolated, so the role of CX3CR1 in NK cells remained unknown. We isolated the CX_3_CR1^+^NK cells from human HCC tissues to better reflect the real tumor environment. Our results Together, these findings suggested that loss of CX_3_CL1 contributes to the absence of CX_3_CR1^+^NK cells during HCC development.

We also demonstrated that these CX_3_CL1- mediated effects likely involve the activation of STAT3 signaling in NK cells. Although a growing body of literature has linked NK cell dysfunction and cancer 26-29, the regulation of NK cell migration to the tumor nest has remained poorly understood. The tumor microenvironment can contain a diverse repertoire of chemokines and cytokines, with tumors capable of transforming their chemokine patterns to hinder NK recruitment and infiltration. While a variety of cells are recruited to tumor sites 30-32, NK cells are particularly suppressed during tumor development 33. Basic research and clinical studies have indicated that the tumor microenvironment is often configured to block NK cell recruitment and function. However, if not blocked, NK cells can function as robust effectors against tumors 34-36. Presently, we isolated CX_3_CR1^+^NK cells from HCC tissues and used murine models to confirm their vital role in HCC progression. We also validated these cells as integral components of the miR-561-5p/CX_3_CL1 signaling regulation of pulmonary metastasis. These experiments demonstrated that the tumor microenvironment can be altered through a series of events initiated by changes in miR-561-5p expression.

Our evidence of HCC-specific expression patterns and pro-metastatic function nominated miR-561-5p as an independent prognostic biomarker and a potential intervention target. We also observed that miR-561-5p, CX_3_CL1, and CX_3_CR1^+^NK cells levels can serve as independent indicators of HCC prognosis, with the combination of these factors providing a more sensitive independent prognostic measure. Thus, it is important to further explore the antitumor effects of these factors *in vivo*. Accumulating evidence has indicated the contribution of the CX_3_CL1-CX_3_CR1 axis to the pathogenesis of many disorders including cancer 37, 38, inflammatory 39 and diabetes 40. We cannot investigate all the CX_3_CR1 expressing immune cells in the tumor.

In summary, this study elucidated a miR-561- 5p/CX_3_CL1/CX_3_CR1^+^NK cells axis that delineates mechanisms underlying HCC pulmonary metastasis. In addition, miR-561-5p, CX_3_CL1, and CX_3_CR1^+^NK cells can be used to gauge the prognosis of HCC patients undergoing curative resection. Moreover, components of this signaling axis represent potential immunotherapy targets to combat immune escape strategies in HCC.

## Supplementary Material

Supplementary figures, materials and methods, and tables.

Supplementary table.

## Figures and Tables

**Table 1:** Univariate and multivariate analyses of prognostic factors with TTR and OS in HCC (n = 242)

Variables	TTR			OS	
	HR (95%CI)	P		HR (95%CI)	P
**Univariate analysis**					
Age, years (≤50 vs. >50)	0.830(0.583-1.180)	0.299		0.986(0.673-1.446)	0.944
Sex (female vs. male)	1.770(0.898-3.486)	0.099		1.854(0.862-3.988)	0.114
HBsAg (negative vs. positive)	1.229(0.788-1.917)	0.363		1.097(0.682-1.766)	0.703
AFP, ng/mL (≤20 vs. >20)	1.722(1.158-2.562)	**0.007**		1.570(1.026-2.402)	**0.038**
γ-GT, U/L (≤54 vs. >54)	1.448(0.987-2.124)	0.058		1.602(1.047-2.450)	**0.030**
Liver cirrhosis (no vs. yes)	1.296(0.910-1.847)	0.151		1.521(1.032-2.242)	**0.034**
Tumor size, cm (≤5 vs. >5)	1.469(1.036-2.082)	**0.031**		2.109(1.441-3.086)	**0.000**
Tumor number (single vs. multiple)	1.693(1.085-2.644)	**0.021**		1.142(0.681-1.916)	0.615
Microvascular invasion (no vs. yes)	1.535(1.073-2.196)	**0.019**		2.013(1.349-3.002)	**0.001**
Tumor encapsulation (complete vs. none)	1.337(0.943-1.896)	0.103		1.512(1.039-2.202)	**0.031**
Tumor differentiation (I+II vs. II+IV)	1.463(1.000-2.141)	**0.050**		1.984(1.342-2.931)	**0.001**
TNM stage (I vs. II III)	1.501(1.023-2.203)	**0.038**		2.414(1.511-3.856)	**0.000**
MiR-561-5p (low vs. high)	2.281(1.593-3.265)	**0.000**		2.604(1.750-3.875)	**0.000**
CX_3_CL1 (low vs. high)	0.400(0.278-0.575)	**0.000**		0.419(0.283-0.621)	**0.000**
CX_3_CR1+NK (low vs. high)	0.558(0.392-0.795)	**0.001**		0.544(0.371-0.798)	**0.002**
Combination miR-561-5p, CX_3_CL1 and CX3CR1+NK					
I vs. II	1.928(1.182-3.146)	**0.007**		1.971(1.140-3.140)	**0.013**
II vs. III	1.928(1.182-3.146)	**0.009**		1.971(1.140-3.410)	**0.015**
I vs. III	3.867(2.426-6.165)	**0.000**		3.987(2.404-6.613)	**0.000**
**Multivariate analysis**					
AFP, ng/mL (≤20 vs. >20)	1.342(0.889-2.028)	0.162		1.014(0.650-1.583)	0.951
γ-GT, U/L (≤54 vs. >54)	NA	NA		1.437(0.921-2.243)	0.110
Liver cirrhosis (no vs. yes)	NA	NA		1.283(0.861-1.911)	0.220
Tumor size, cm (≤5 vs. >5)	1.576(1.098-2.261)	**0.014**		2.124(1.409-3.200)	**0.000**
Tumor number (single vs. multiple)	1.77(1.113-2.816)	**0.016**		NA	NA
Microvascular invasion (no vs. yes)	1.469(1.015-2.127)	**0.042**		1.874(1.228-2.858)	**0.004**
Tumor encapsulation (complete vs. none)	NA	NA		1.369(0.922-2.033)	0.119
Tumor differentiation (I+II vs. II+IV)	1.468(0.989-2.178)	0.056		1.677(1.111-2.533)	**0.014**
miR-561-5p (low vs. high)	2.915(1.787-4.754)	**0.000**		3.659(2.126-6.298)	**0.000**
CX_3_CL1 (low vs. high)	0.355(0.223-0.566)	**0.000**		0.383(0.229-0.639)	**0.000**
CX_3_CR1+NK (low vs. high)	2.003(1.139-3.520)	**0.016**		2.206(1.184-4.112)	**0.013**
Combination miR-561-5p, CX_3_CL1 and CX3CR1+NK					
I vs. II	1.891(1.135-3.151)	**0.014**		1.817(1.025-3.221)	**0.041**
II vs. III	1.811(1.086-3.018)	**0.023**		1.890(1.136-3.144)	**0.014**
I vs. III	4.249(2.645-6.826)	**0.000**		4.380(2.621-7.319)	**0.000**

## Abbreviations

NK cells: natural killer cells
HCC: hepatocellular carcinoma
CX_3_CL1: C-X_3_-C motif ligand 1
miRNA: microRNA
CX_3_CR1: chemokine (C-X_3_-C motif) receptor 1
STAT3: transducer and activator of transcription 3
FAK: focal adhesion kinase
JNK: c-Jun NH-terminal kinase
STAT1: transducer and activator of transcription 1
p38-MAPK: p38 mitogen- activated protein kinase
ERK: extracellular signal regulated kinase
qRT-PCR: Quantitative Polymerase Chain Reaction
OS: overall survival
TTR: time to recurrence

## References

[1] Siegel RL, Miller KD, Jemal A (2018). Cancer statistics, 2018. CA Cancer J Clin.

[2] Llovet JM, Villanueva A, Lachenmayer A, Finn RS (2015). Advances in targeted therapies for hepatocellular carcinoma in the genomic era. Nat Rev Clin Oncol.

[3] Chakravorty SJ, Cockwell P, Girdlestone J, Brooks CJ, Savage CO (2002). Fractalkine expression on human renal tubular epithelial cells: potential role in mononuclear cell adhesion. Clin Exp Immunol.

[4] Hamerman JA, Ogasawara K, Lanier LL (2005). NK cells in innate immunity. Curr Opin Immunol.

[5] Miller JS, Soignier Y, Panoskaltsis-Mortari A, McNearney SA, Yun GH, Fautsch SK (2005). Successful adoptive transfer and in vivo expansion of human haploidentical NK cells in patients with cancer. Blood.

[6] Rubnitz JE, Inaba H, Ribeiro RC, Pounds S, Rooney B, Bell T (2010). NKAML: a pilot study to determine the safety and feasibility of haploidentical natural killer cell transplantation in childhood acute myeloid leukemia. J Clin Oncol.

[7] Otaka R, Takahara M, Ueda S, Nagato T, Kishibe K, Nomura K (2017). Up-regulation of CX3CR1 on tonsillar CD8-positive cells in patients with IgA nephropathy. Hum Immunol.

[8] Hamann I, Unterwalder N, Cardona AE, Meisel C, Zipp F, Ransohoff RM (2011). Analyses of phenotypic and functional characteristics of CX3CR1-expressing natural killer cells. Immunology.

[9] Stromberg A, Olsson K, Dijksterhuis JP, Rullman E, Schulte G, Gustafsson T (2016). CX3CL1-a macrophage chemoattractant induced by a single bout of exercise in human skeletal muscle. Am J Physiol Regul Integr Comp Physiol.

[10] Vitale S, Cambien B, Karimdjee BF, Barthel R, Staccini P, Luci C (2007). Tissue-specific differential antitumour effect of molecular forms of fractalkine in a mouse model of metastatic colon cancer. Gut.

[11] He L, Hannon GJ (2004). MicroRNAs: small RNAs with a big role in gene regulation. Nat Rev Genet.

[12] Zhang Y, Yang P, Wang XF (2014). Microenvironmental regulation of cancer metastasis by miRNAs. Trends Cell Biol.

[13] Bartel DP (2004). MicroRNAs: genomics, biogenesis, mechanism, and function. Cell.

[14] Croce CM (2009). Causes and consequences of microRNA dysregulation in cancer. Nat Rev Genet.

[15] Fang JH, Zhang ZJ, Shang LR, Luo YW, Lin Y, Yuan Y (2018). Hepatoma cell-secreted exosomal microRNA-103 increases vascular permeability and promotes metastasis by targeting junction proteins.

[16] Lv C, Li F, Li X, Tian Y, Zhang Y, Sheng X (2017). MiR-31 promotes mammary stem cell expansion and breast tumorigenesis by suppressing Wnt signaling antagonists. Nat Commun.

[17] Chen EB, Zhou SL, Pang XG, Yin D, Miao PZ, Yang Y (2017). Prostate-derived ETS factor improves prognosis and represses proliferation and invasion in hepatocellular carcinoma. Oncotarget.

[18] Yang P, Li QJ, Feng Y, Zhang Y, Markowitz GJ, Ning S (2012). TGF-beta-miR-34a-CCL22 signaling-induced Treg cell recruitment promotes venous metastases of HBV-positive hepatocellular carcinoma. Cancer cell.

[19] Marelli G, Erreni M, Anselmo A, Taverniti V, Guglielmetti S, Mantovani A (2017). Heme-oxygenase-1 Production by Intestinal CX3CR1(+) Macrophages Helps to Resolve Inflammation and Prevents Carcinogenesis. Cancer Res.

[20] Sun C, Sun HY, Xiao WH, Zhang C, Tian ZG (2015). Natural killer cell dysfunction in hepatocellular carcinoma and NK cell-based immunotherapy. Acta Pharmacol Sin.

[21] Li M, Yang Y, He ZX, Zhou ZW, Yang T, Guo P (2014). MicroRNA-561 promotes acetaminophen-induced hepatotoxicity in HepG2 cells and primary human hepatocytes through downregulation of the nuclear receptor corepressor dosage-sensitive sex-reversal adrenal hypoplasia congenital critical region on the X chromosome, gene 1 (DAX-1). Drug Metab Dispos.

[22] Zhou SL, Hu ZQ, Zhou ZJ, Dai Z, Wang Z, Cao Y (2016). miR-28-5p-IL-34-macrophage feedback loop modulates hepatocellular carcinoma metastasis. Hepatology.

[23] Cacalano NA (2016). Regulation of Natural Killer Cell Function by STAT3. Front Immunol.

[24] Gorini S, Callegari G, Romagnoli G, Mammi C, Mavilio D, Rosano G (2010). ATP secreted by endothelial cells blocks CX(3)CL 1-elicited natural killer cell chemotaxis and cytotoxicity via P2Y(1)(1) receptor activation. Blood.

[25] Aoyama T, Inokuchi S, Brenner DA, Seki E (2010). CX3CL1-CX3CR1 interaction prevents carbon tetrachloride-induced liver inflammation and fibrosis in mice. Hepatology.

[26] Ferrari de Andrade L, Tay RE, Pan D, Luoma AM, Ito Y, Badrinath S (2018). Antibody-mediated inhibition of MICA and MICB shedding promotes NK cell-driven tumor immunity. Science.

[27] Bottcher JP, Bonavita E, Chakravarty P, Blees H, Cabeza-Cabrerizo M, Sammicheli S (2018). NK Cells Stimulate Recruitment of cDC1 into the Tumor Microenvironment Promoting Cancer Immune Control. Cell.

[28] Wagner J, Kline CL, Zhou L, Campbell KS, MacFarlane AW, Olszanski AJ (2018). Dose intensification of TRAIL-inducing ONC201 inhibits metastasis and promotes intratumoral NK cell recruitment.

[29] Lopez-Soto A, Gonzalez S, Smyth MJ, Galluzzi L (2017). Control of Metastasis by NK Cells. Cancer Cell.

[30] Morandi F, Ferretti E, Castriconi R, Dondero A, Petretto A, Bottino C (2011). Soluble HLA-G dampens CD94/NKG2A expression and function and differentially modulates chemotaxis and cytokine and chemokine secretion in CD56bright and CD56dim NK cells. Blood.

[31] Pallandre JR, Krzewski K, Bedel R, Ryffel B, Caignard A, Rohrlich PS (2008). Dendritic cell and natural killer cell cross-talk: a pivotal role of CX3CL1 in NK cytoskeleton organization and activation. Blood.

[32] Barlic J, Sechler JM, Murphy PM (2003). IL-15 and IL-2 oppositely regulate expression of the chemokine receptor CX3CR1. Blood.

[33] Chockley PJ, Chen J, Chen G, Beer DG, Standiford TJ, Keshamouni VG (2018). Epithelial-mesenchymal transition leads to NK cell-mediated metastasis-specific immunosurveillance in lung cancer. J Clin Invest.

[34] Barrow AD, Edeling MA, Trifonov V, Luo J, Goyal P, Bohl B (2018). Natural Killer Cells Control Tumor Growth by Sensing a Growth Factor. Cell.

[35] Freud AG, Mundy-Bosse BL, Yu J, Caligiuri MA (2017). The Broad Spectrum of Human Natural Killer Cell Diversity. Immunity.

[36] Wagner JA, Rosario M, Romee R, Berrien-Elliott MM, Schneider SE, Leong JW (2017). CD56bright NK cells exhibit potent antitumor responses following IL-15 priming. J Clin Invest.

[37] Xin H, Kikuchi T, Andarini S, Ohkouchi S, Suzuki T, Nukiwa T (2005). Antitumor immune response by CX3CL1 fractalkine gene transfer depends on both NK and T cells. Eur J Immunol.

[38] Park MH, Lee JS, Yoon JH (2012). High expression of CX3CL1 by tumor cells correlates with a good prognosis and increased tumor-infiltrating CD8+ T cells, natural killer cells, and dendritic cells in breast carcinoma. Journal of surgical oncology.

[39] Moore KJ, Sheedy FJ, Fisher EA (2013). Macrophages in atherosclerosis: a dynamic balance. Nat Rev Immunol.

[40] Riopel M, Seo JB, Bandyopadhyay GK, Li P, Wollam J, Chung H (2018). Chronic fractalkine administration improves glucose tolerance and pancreatic endocrine function. J Clin Invest.

