# Supplementary material for: The miR-561-5p/CX3CL1 Signaling Axis Regulates Pulmonary Metastasis in Hepatocellular Carcinoma Involving CX3CR1+ Natural Killer Cells Infiltration
Source: Theranostics. 2019 Jul 9;9(16):4779–94. doi: 10.7150/thno.32543 (PMC6643446; doi:10.7150/thno.32543)
Supplement: Supplementary file 1 — Supplementary figures, materials and methods, and tables. [file thnov09p4779s1.pdf]

Supplementary Figure

Supplementary Figure S1

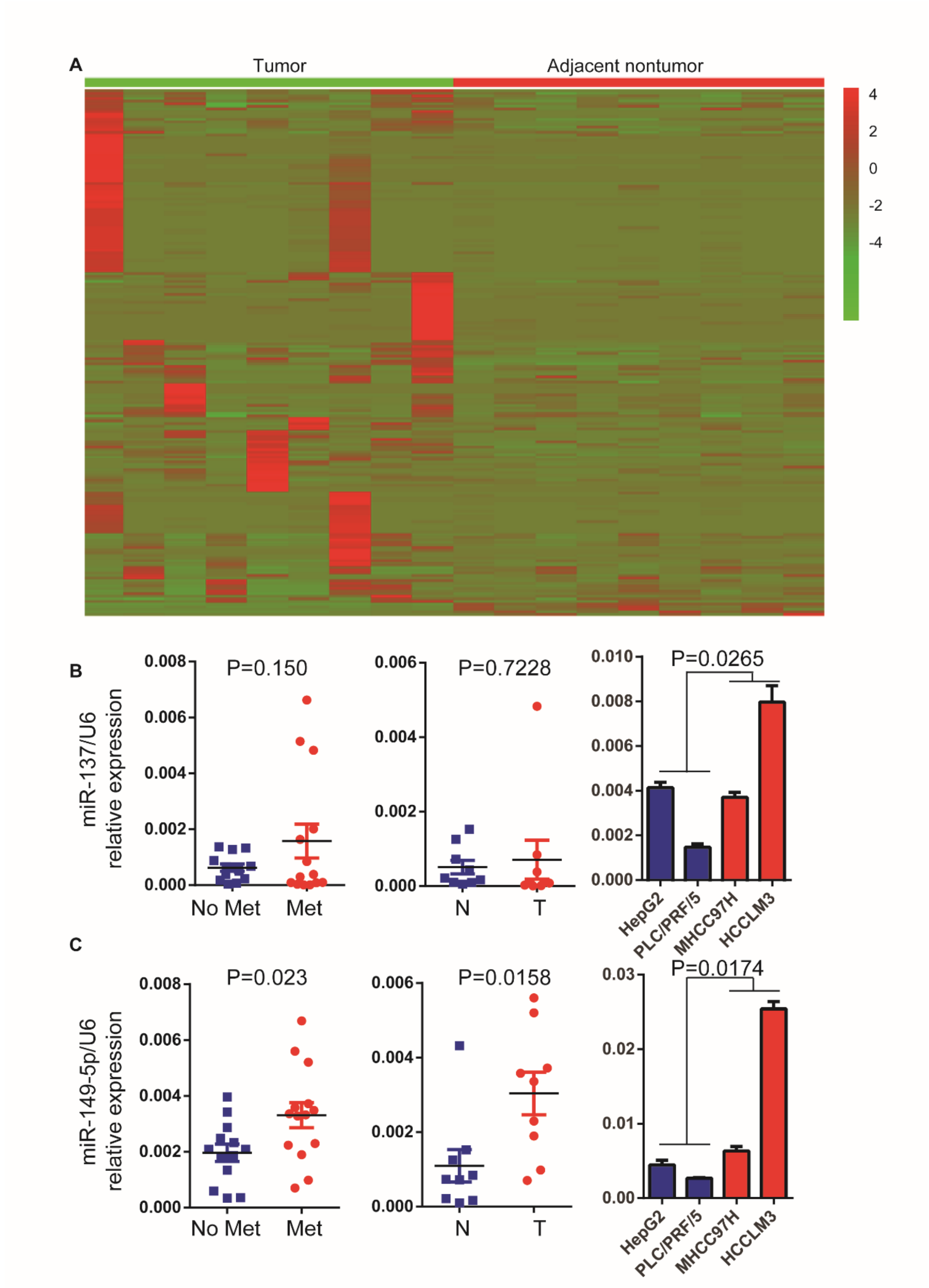

Supplementary Figure S1. MiR-137 and miR-149 expression was upregulated and positively correlated with pulmonary metastasis in HCCs. (A) A heat map clustering

of miRNAs with expression patterns in the tumor compared to adjacent non-tumor tissues in HCCs. (B-C) qRT-PCR revealed that miR-137 and miR-149 expression was significantly increased in HCC tumor with metastasis (Met/T) or high metastatic potential HCC cell lines, when compared to HCC without metastasis (No Met) or corresponding adjacent nontumor tissues (N) or low metastatic potential HCC cell lines.

Supplementary Figure S2

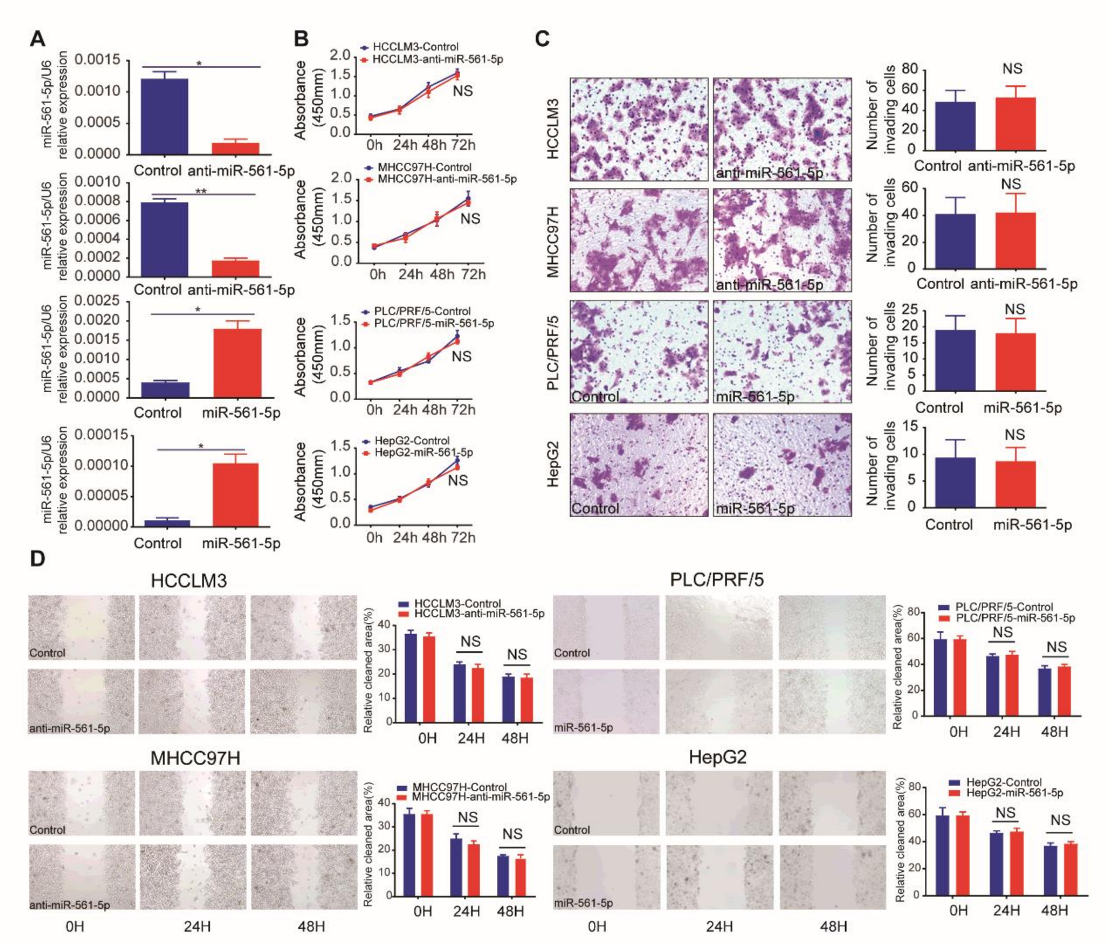

Supplementary Figure S2. MiR-561-5p had no effect on cancer cell proliferation, migration and invasion. (A) qRT-PCR confirmed the results of miR-561-5p or anti-miR-561-5p lentiviral vector transfection into human HCC cell lines. (B) Cell proliferation measured by CCK8 assay showed no significant difference between stably transfected and parent HCC cells. (C) Invasive behaviour was evaluated by transwell Matrigel invasion assays. (D) Cell monolayers were wounded and monitored at 0, 24 and 48 hours for wound channel closure. The cleared area was measured and plotted as the percentage of the original area at 0 hours. Data shown are mean±SD from three independent experiments， each performed in triplicate. (N.S., not significance; Student’s t-tests).

Supplementary Figure S3

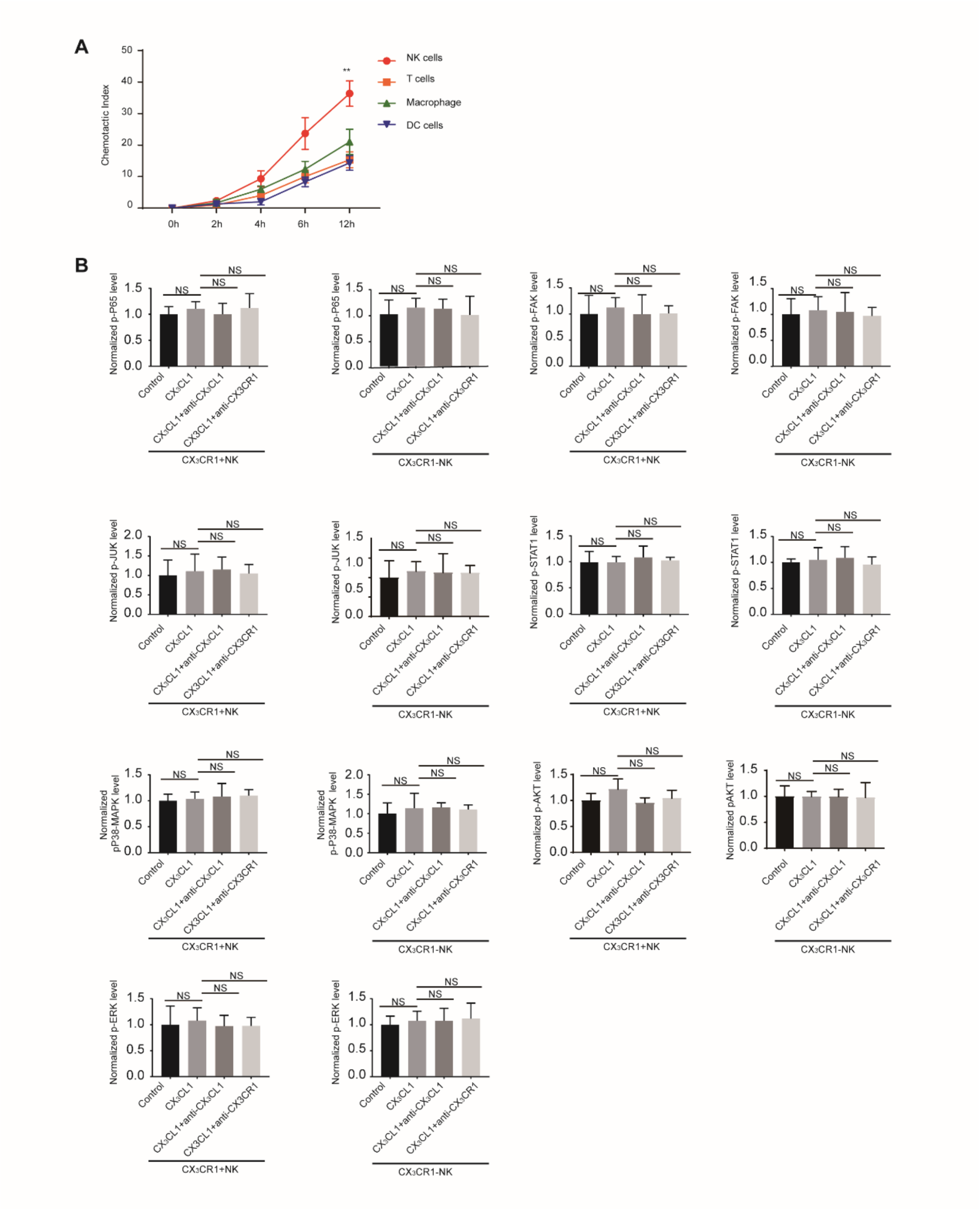

Supplementary Figure S3. CX3CR$^+$NK cells were the strongest effector cells by STAT3 activation upon CX$_3$CL1. (A) The chemotactic assay showed that with the treatment of CX$_3$CL1, the migration ability of NK cells is strongest compared to macrophage, T cells, and DC cells. (B) Western blotting showed the phosphorylation level NFKB, FAK, JUK, STAT1, P38-MAPK, AKT and ERK in both CX$_3$CR1$^+$ and

CX_3CR1^-NK cells did not change. Data shown are mean±SD from three independent experiments， each performed in triplicate. (N.S., no significance; **P<0.01. Student's t-tests).

Supplementary Figure S4

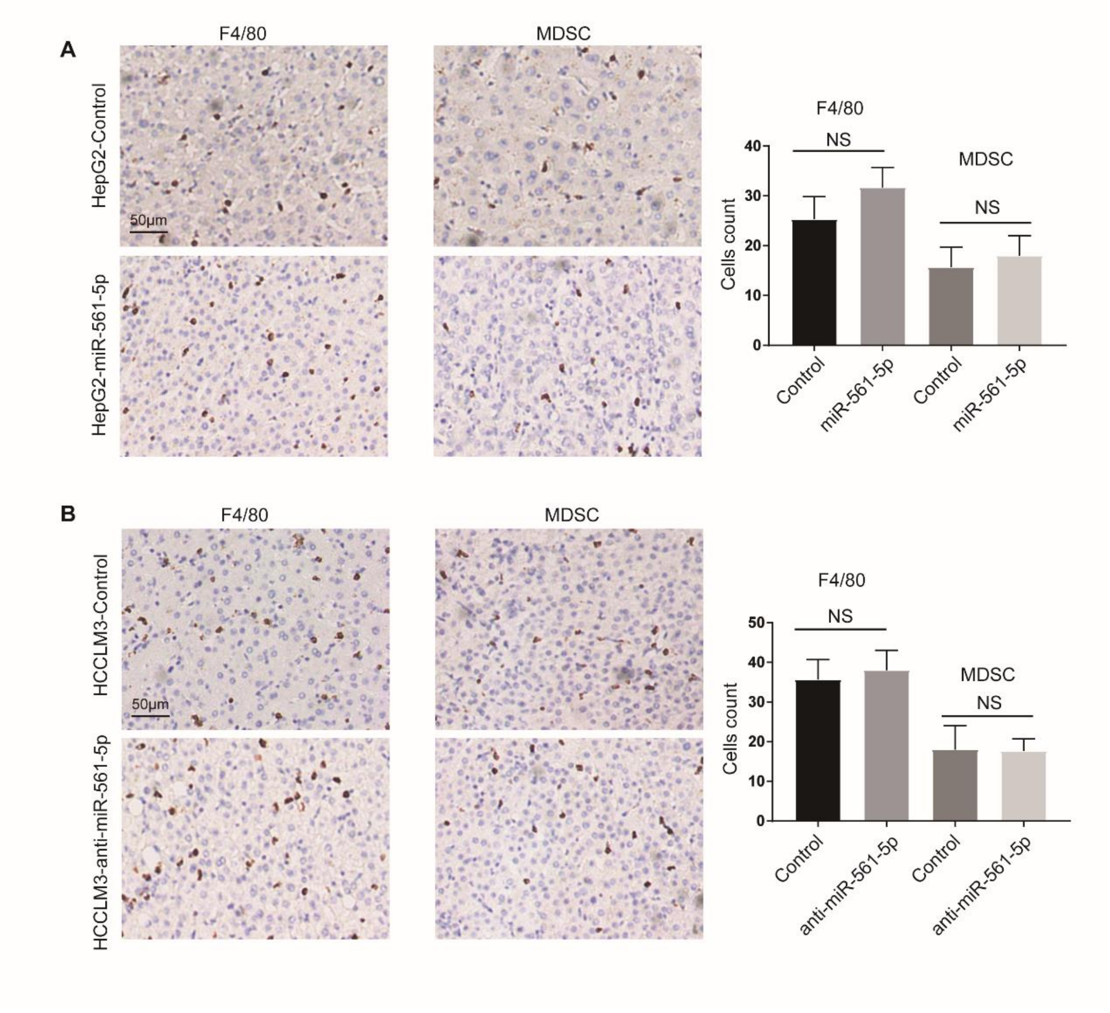

Supplementary Figure S4. MiR-561-5p does not affect the infiltration of monocytes and myeloid derived suppressor cells into the tumor bed. (A) Representative images of monocyte and myeloid derived suppressor cells in HepG2-miR-561-5p and control groups. (B) Representative images of monocyte and myeloid derived suppressor cells in HCCLM3-anti-miR-561-5p and control groups. Data shown are mean±SD from three independent experiments. (N.S., no significance. Student's t-tests).

Supplementary Figure S5

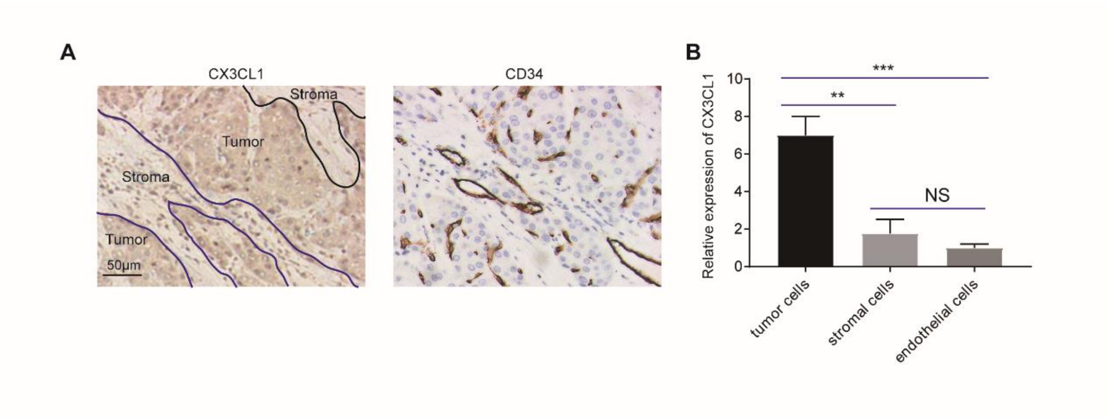

Supplementary Figure S5. The main source of CX_3CL1 was tumor cells. The main source of CX_3CL1 in tumor tissues was tumor cells (A, left panel), compared with the endothelial cells (A, right panel) and stromal cells (A, left panel). (B) Relative expression of CX_3CL1 was shown in three different cell regions. (N.S., no significance; **, P<0.01, ***, P<0.001. Student's t-tests).

Supplementary Figure S6

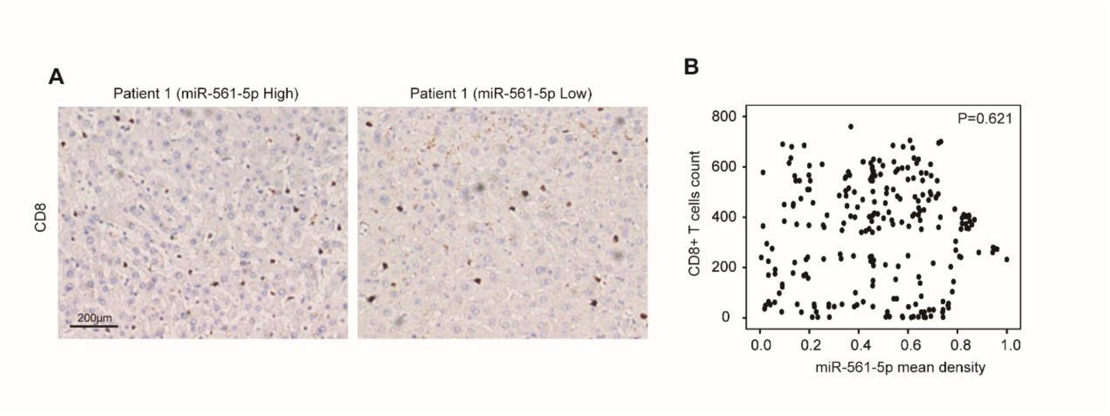

Supplementary Figure S6. The numbers of CD8+ T cells in tumor tissues was not associated with miR-561-5p expression. (A) Representative images of $CD8^+$ T cells were shown in TMA. Scale bar, 200x, 50μm. (B) The number of $CD8^+$ T cells was not associated with the expression of miR-561-5p in clinical samples (P=0.621).

Supplementary Materials and Methods

Vectors and Cell Transfections

The following miRNA vectors were purchased from Shanghai GeneChem Co.,Ltd. miR-561-5p expression and the control vector for miR-561-5p (Ubi-Luc-MCS-IRES-Puromycin); miR-561-5p inhibitor and the negative control for the miR-561-5p in miR-561-5p inhibitor (U6-MCS-Ubi-Luc). Ubi-Luc-MCS-IRES-Puromycin-miR-561-5p was transfected into HCC cells with lower metastatic potential (HepG2 and PLC/PRF/5) and mU6-MCS-Ubi-anti-miR-561-5p was transfected into higher metastatic HCC cells (HCCLM97H and HCCLM3).

Enzyme-linked immunosorbent assay (ELISA)

We determined the level of cytokines/chemokines in cell culture supernatants by using the corresponding Fractalkine (CX_3CL1) Human SimpleStep ELISA Kit (ab192145) in accordance with the accordance with the manufacturer's instructions. Briefly, we added 50 μL of sample to each well and incubated the plates for 1 h as RT. After washing, we determined immunoreactivity by adding substrate solution, and the absorbance was determined using a Microplate Spectrophotometer (Bio-Rad). A curve of the absorbance versus the concentrations of cytokines/chemokines in the standard wells was plotted.

In situ hybridization

TMA slides samples with 4 μm thick, hatching at 60°C for 1 h, deparaffinized with xylene, and rehydrated with a series of graded alcohol washes. Slides were then washed with RNase-free PBS (three times), digested with 8 mg/ml pepsin at 3°C for 10 min,

washed, and then graded alcohol dehydration. Slides were hybridized at 40°C overnight with 50 nm locked nucleic acid (LNA)-modified DIG-labeled probes for miR-561-5p (Exiqon). After stringency washes (5×, 1×, 0.2 × SSC), slides then were placed in blocking buffer for 30 min at room temperature followed by overnight incubation at 4°C in alkaline phosphatase conjugated anti-DIG Fab fragment solution. Antibody signal was stained with NBT and BCIP substrate (Roche, Mannheim, Germany) and then the nuclei of cells were stained by Nuclear Fast Red

NK cells isolation

For NK cells isolation, peripheral blood from the healthy donors were isolated by magnetic-activated cell sorting (MACS) using a direct CD56 Isolation Kit (Miltenyi Biotec) in accordance with the manufacturer's instructions.

For CX_3CR1^+/CX_3CR1^-NK cell isolation, peripheral blood samples were collected, CX_3CR1^+/CX_3CR1^-NK isolated by FACS using CX_3CR1 antibody and CD56 antibody, according to the manufacturer's instructions.

Chemotaxis assay

We assayed cell chemotaxis using a Transwell system (Coring) with 5μm polycarbonate membranes. CX_3CR1^+ or CX_3CR1^-NK cells suspended in RPMI 1640 containing 2% FBS (1X105 cells/100μl) were added to the upper wells and incubated for 24 hours at 37°C, and 5%CO_2. Human CX_3CL1 at various concentration, or supernatants from cancer cells derived from different sources, with or without control Ab, c\anti-CX_3CL1 or anti-CX_3CR1 antibodies were added to the lower chamber. We collected them in Neubauer chambers; NK cells that migrated and attached to the lower surface of the

transwell membrane were fixed with 4% paraformaldehyde, followed with 0.5% crystal violet (Sigma-Aldrich) staining and then subjected to phase-contrast microscopy images under 200X objective, and the negative control comprised cells that migrated toward PRMI 1640 alone. The relative chemotactic index represents the number of cells that migrated toward the chemoattractant in the lower chamber compared with the negative control

NK cytotoxicity assay

NK cell cytotoxic activity against the tumor cells was determined by measuring the amount of lactate dehydrogenase (LDH) released from the target cells. A commercial LDH cytotoxicity kit (Beyotime) was used according to the manufacturer's instructions. The maximum LDH release was determined by lysing target cells for 30 min using the lysis buffer provided with the assay and measuring LDH in the culture medium. Absorbance for the colorimetric reaction was measured at a wavelength of 490 nm, with a reference wavelength of 655 nm, using a Model 550 microplate reader (Bio-Rad, Hercules, CA, USA). The specific lysis for each effector to target cell (E: T) ratio was calculated with the following formula: % specific lysis = [(experimental release − spontaneous release)/ (maximum release − spontaneous release)] × 100.

Tissue microarray and immunohistochemistry

Pairs of 242 specimens diagnosed as HCC plus normal tissue adjacent to the tumors were formalin-fixed, paraffin-embedded, then stored at -20°C. For analysis, the tissue sections were dewaxed, followed by rehydration using dimethylbenzene and hydrous ethanol. Endogenous peroxidase activity was blocked by incubation with 3% hydrogen

peroxide for 15 min. Sections submerged in 10 mmol/L citrate at pH 6 were microwaved on high power for 7 min, followed by washing with phosphate-buffered saline (PBS) for 3 min. The sections were subsequently incubated overnight at 4°C with antibody with blocking solution. This was followed by three PBS washes, after which the slides were exposed to secondary antibody at room temperature for 1 h.

Immunohistochemistry staining was conducted using the avidin-biotin complex method. A position stain was developed using a-diaminobezidine. Data were collected regarding signal intensity and percentage of staining. When the primary antibody staining was unclear, the cells were categorized as high or low by comparing them with cells on the corresponding benign (adjacent) tissue slides. Negative controls were analyzed in a similar manner, except for the use of primary antibodies.

Evaluation of immunohistochemical variables

Immunohistochemical staining was assessed by three independent investigators who were blinded to patient characteristics, and discrepancies were resolved by consensus. The density of positive staining was captured with the use of a computerized image system composed of a Leica CCD camera DFC420 connected to a Leica DM IRF2 microscope (Leica Microsystems Imaging Solutions Ltd, Cambridge, United Kingdom). Under 200X magnification, photographs of three representative fields were captured by the Leica QWin Plus v3 software. Photographs of identical settings were used for each photograph. The density of miR-561-5p, CX_3CL1 and CX_3CR1 was measured by Image-Pro Plus v6.2 software (Media Cybernetics Inc, Bethesda, MD). For the reading of each antibody staining, a uniform setting for all the slides was applied. Integrated

optical density of all the positive of miR-561-5p, CX_3CL1 and CX_3CR1 in each photograph was measured, and its ratio to total area of each photograph was calculated as miR-561-5p, CX_3CL1 and CX_3CR1 density. CD56-postive areas in the photographs were assessed by the Leica Qwin Plus.

Median values were used as a cut-off in subsequent analyses unless specified.

RNA isolation, qRT-PCR, and western blot analysis

Total RNA extraction and isolation were performed using Trizol Reagent (Invitrogen, USA). Reverse transcription of 2 μg total RNA was performed using PrimeScript PT Reagent Kit (TaKaRa, Japan). mRNA expression levels were measured by qRT-PCR utilizing SYBR Premix Ex Taq II (TaKaRa,Japan). PCR amplification involved an initial 2-min step at 95°C, then 40 cycles of the following: 15 sec at 95°C, 30 sec at 60°C, and 30 sec at 70°C. Glyceraldehyde-3-phosphate dehydrogenase (GAPDH) was employed as an endogenous control to normalize the quantity of RNA among samples. Cells at 90% confluence were washed three times with ice-cold PBS, then extracted in RIPA buffer containing protease inhibitors. Total protein was extracted according to the instructions. All samples were added to 5X loading buffer and boiled for 5 min. Protein (50 μg) extracted from each sample was resolved by 10% sodium dodecyl sulfate-polyacrylamide gel electrophoresis (SDS-PAGE). These extracted samples were transferred to a polyvinylidene difluoride membrane (Millipore, USA), which was blocked by incubation at room temperature for 1 h with TBST buffer (20 mM Tris/HCl, pH 7.5; 0.137M NaCI; 0.05% Tween-20) containing 5% nonfat skim milk. The

membrane was then probed with primary antibody (Abcam, USA) diluted 1:200 with TBST buffer, followed by addition of a secondary antibody. Protein was detected using enhanced chemiluminescence. GAPDH (Cell Signaling Technology, USA) was used as a loading control. Each experiment was conducted three times.

Cell proliferation assay, cell migration, and cell invasion assay

Using a 96-well culture plate, 1000 cells were plated onto each well and incubated for 0, 24, 48, and 72 h at 37°C under 5% carbon dioxide. At each time, 10μL of cell counting kit-8 solution (Dojindo, Japan) were added, and absorbance at 450nm was determined using an Infinite 200 spectrometer. We performed each assay in triplicate and conducted three independent trials.

Cell migration was examined by the scratch wound assay. After culturing for 2 days to establish a tight monolayer, the cells were deprived of serum for 16 h. The monolayer was then wounded using a 10-μL plastic pipette tip. The cells were washed twice to remove cell debris, followed by incubation with standard culture medium (containing serum) at 37°C. Cells migrating to the wound front were photographed after 24 and 48h using an inverted microscope (Leica, Hesse, Germany). Migration capacity was quantified by measuring the percent open area.

After suspended in 150 μL DMEM medium with 1% FBS, treated and untreated (control) HCC cells were seeded in triplicate onto the upper chamber of a transwell insert, with 50,000 cells per well. Medium containing 10% FBS was placed in the lower chamber to function as a chemoattractant. After 48 h incubation, upper chamber cells were extracted by scraping, and those cells left on the lower insert surface underwent

fixation with 4% paraformaldehyde, followed by 10 min of crystal violet staining. The numbers of cells in 10 random microscope fields (magnification, 200X) were counted. Error bars in Figure represent the standard deviation of three distinct data sets.

Flow cytometry for CX_3CR1^+NK cells analysis

Cells were harvested after 48 h, then fixed and dehydrated for 24 h using 70% ethanol at -20ºC. After washing twice with PBS, the cells were resuspended in 500 μL solution containing 0.5 mg/mL propidium iodide and 1mg/mL RNase A (Sigma-Aldrich, USA). This solution was kept in the dark before analysis. After 48 h, the cells were again harvested and washed twice with PBS. They were subsequently centrifuged, then stained simultaneously with Alexa Flur488 Annexin V and PI (Life Technology, USA). Stained cells underwent flow cytometry.

Reagents and antibodies

The following antibodies were used: anti-CX_3CL1 (ab25088, Abcam, USA), anti-CX_3CR1 (ab8021, Abcam, USA), anti-CD56 (ab9272, Abcam, USA), anti-STAT3 (CST#9139, Cell Signaling Technology, USA), anti-phospho-STAT3(CST#9145, Cell Signaling Technology, USA), anti-P65(CST#8242, Cell Signaling Technology, USA), anti- phospho-P65(CST#3033, Cell Signaling Technology, USA), anti-FAK(CST#71433, Cell Signaling Technology, USA), anti-phospho-FAK(CST#8556, Cell Signaling Technology, USA), anti-JNK(CST#3708, Cell Signaling Technology, USA), anti-phospho-JNK(CST#9255, Cell Signaling Technology, USA), anti-STAT1(CST#14994, Cell Signaling Technology, USA), anti-phospho-STAT1(CST#9167, anti-AKT1(CST#75692, Cell Signaling Technology, USA), anti-

phospho-AKT1(CST#9018, Cell Signaling Technology, USA), anti-P38(CST#8690, Cell Signaling Technology, USA), anti-phospho-P38(CST#4511, Cell Signaling Technology, USA), anti-ERK(CST#4370, Cell Signaling Technology, USA), anti-phospho-ERK(CST#5683, Cell Signaling Technology, USA), and GAPDH (CST#5174, Cell Signaling Technology, USA).

miR-137, miR-149-5p, and miR-561-5p were purchased from Qiagen (Qiagen, Germany). CX_3CL1 (forward prrimer:5'- TCCTTATCACTCCTGTCCCTGACG-3' and reverse primer:5'- TGTCCCTGGAAGGTGGAGAATG-3'), and universal probe the glyceraldehyde-3-phosphate dehydrogenase (GAPDH) (forward prrimer 5'-AGCCACATCGCTCAGACAC-3' and reverse primer 5'-GCCCAATACGACCAAA TCC-3') were purchased from the Sangon Biotech (Shanghai) Co., Ltd.

Statistical analysis

Two-sided paired Student's t-tests were used to compare quantitative samples data. OS and tumor-free survival were estimated using the Kaplan-Meier method. The log-rank test was employed to assess survival differences between patients with miR-561-5p, CX_3CL1 and CD56. Chi-square or Fisher exact tests were used for categorical data. Univariate and multivariate analyses were based on Cox proportional hazard regression models. Data are presented as mean±standard deviation. $P \leq 0.05$ was considered statistically significant. SPSS software for Windows (version 21, USA) was used for all analyses.

Supplementary Table S1. Characteristics of study participants in the RNA-sequencing.

variable	Metastasis count (n=14)	No metastasis count (n=13)	P value
Age, years			0.78
Mean	55	54	
SD	10	9	
Sex			
Male	11	12	0.60
Female	3	1	
AFP			0.68
≤400ng/mL	9	10	
>400ng/mL	5	3	
GGT			0.90
≤54U/L	10	9	
>54U/L	4	4	
Tumor size			0.18
≤5cm	5	8	
>5cm	9	5	
Liver cirrhosis			0.67
Yes	10	8	
No	3	5	
Tumor encapsulation			
Complete	10	9	1.00
None	4	4	
Tumor differentiation			1.00
I+II	9	9	
III+IV	5	4	
TNM stage			0.18
IA	5	8	
IB	9	5	
Microvascular invasion			1.00
Yes	0	0	
No	14	13	

Supplementary Table S2. Changed expression level of cytokines /chemokines in HCC cells upon miR-28-5p or anti-miR-28-5p treatment in four HCC cell lines.

PLC/PRF/5		HepG2		MHCC97H		HCCLM3	
**Control vs miR-561-5p**	**Fold change**	**Control vs miR-561-5p**	**Fold change**	**anti-miR-561-5p vs Control**	**Fold change**	**anti-miR-561-5p vs Control**	**Fold change**
IL17A	4138.04	TNFRSF11B	3867.53	CXCL9	335.37	CCL22	39.65
TGFB2	4023.81	CCL18	2006.19	CXCL11	170.78	**CX3CL1**	**14.07**
IL7	328.25	CCL22	1362.02	XCL1	120.52	CXCL9	9.33
CCL2	218.00	IL4	1114.07	IFNA2	81.78	TNF	7.33
IL9	35.69	IFNG	642.52	CCL2	75.19	CCL19	6.56
IL1A	11.12	LTA	398.14	IL21	62.19	IL9	4.44
BMP7	4.66	MSTN	334.30	LTA	40.61	HGDC	3.23
IL5	2.63	FASLG	321.13	CCL21	26.41	IFNG	3.01
CCL8	2.23	CXCL12	310.93	TNFRSF11B	23.83	CCL13	2.79
IL21	2.10	CCL3	202.57	CCL18	18.18	IL10	2.11
BMP2	1.72	CCL2	110.33	**CX3CL1**	**11.46**	BMP7	2.06
**CX3CL1**	**1.30**	IL6	84.72	LTB	10.62	MSTN	1.87
IL1RN	1.18	CXCL9	55.84	ADIPOQ	9.60	IL23A	1.49
MSTN	1.14	CCL21	21.09	B2M	9.39	CSF3	1.22
GAPDH	1.00	IL21	14.34	IL1A	9.07	CCL8	1.22
		IL16	13.63	CXCL2	7.99	ACTB	1.19
		NODAL	12.80	CNTF	6.96	IL1B	1.04
		IFNA2	9.96	CSF2	6.81	GPI	1.00
		PPBP	7.88	CXCL1	6.14		
		TNFSF13B	4.12	IL8	5.83		
		CSF2	3.52	SPP1	5.48		
		CCL24	3.39	BMP2	5.44		
		IL17A	3.31	CCL20	5.36		
		IL24	3.25	C5	5.29		
		IL1B	3.01	CXCL5	5.10		
		IL1A	2.73	IL1B	5.01		
		CNTF	2.64	IL18	4.70		
		BMP6	2.35	IL6	4.70		

		TNFSF11	2.08	CSF3	4.09		
		CCL13	1.77	TNFSF13B	3.93		
		ADIPOQ	1.69	TGFB2	3.87		
		**CX3CL1**	**1.53**	IL24	3.68		
		CCL1	1.51	IL15	2.97		
		CCL8	1.48	LIF	2.58		
		CCL19	1.35	IL12A	2.46		
		IL5	1.23	CCL24	2.43		
		TGFB2	1.13	VEGFA	2.34		
			1.00	GPI	2.18		
				BMP4	2.17		
				TNFSF10	2.09		
				RPLP0	1.96		
				CXCL16	1.93		
				HPRT1	1.86		
				ACTB	1.84		
				MIF	1.79		
				IL1RN	1.61		
				RTC	1.56		
				CCL5	1.44		
				IL23A	1.41		
				IL11	1.41		
				CCL7	1.40		
				CSF1	1.23		
				RTC	1.19		
				BMP6	1.02		
				HGDC	1.01		
